# The 1996 North American Interagency Intercomparison of Ultraviolet Monitoring Spectroradiometers

**DOI:** 10.6028/jres.103.028

**Published:** 1998-10-01

**Authors:** Edward Early, Ambler Thompson, Carol Johnson, John DeLuisi, Patrick Disterhoft, David Wardle, Edmund Wu, Wanfeng Mou, James Ehramjian, John Tusson, Tanya Mestechkina, Mark Beaubian, James Gibson, Douglass Hayes

**Affiliations:** National Institute of Standards and Technology, Gaithersburg, MD 20899-0001 USA; National Oceanic and Atmospheric Administration, R/E/ARx1, 325 Broadway, Boulder, CO 80303 USA; Atmospheric Environment Service, Environment Canada, 4905 Dufferin Street, Toronto, ON M3H 5T4, Canada; Dept. of Physics and Astronomy, University of Georgia, Athens, GA 30602 USA; Biospherical Instruments Inc., 5340 Riley Street, San Diego, CA 92110-2621 USA; Yankee Environmental Systems, Inc., Airport Industrial Park Turner Falls, MA 01376 USA; USDA UV-B Radiation Monitoring Program, Natural Resource Ecology Laboratory, Colorado State University, Fort Collins, CO 880523, USA; Smithsonian Environmental Research Center of the Smithsonian Institution, P.O. Box 28, Edgewater, MD 21037 USA

**Keywords:** environmental monitoring, intercomparison, solar ultraviolet, spectroradiometers

## Abstract

Concern over stratospheric ozone depletion has prompted several government agencies in North America to establish networks of spectroradiometers for monitoring solar ultraviolet irradiance at the surface of the Earth. To assess the ability of spectroradiometers to accurately measure solar ultraviolet irradiance, and to compare the results between instruments of different monitoring networks, the third North American Interagency Intercomparison of Ultraviolet Monitoring Spectroradiometers was held June 17–25, 1996 at Table Mountain outside Boulder, Colorado, USA. This Intercomparison was coordinated by the National Institute of Standards and Technology (NIST) and the National Oceanic and Atmospheric Administration (NOAA). Participating agencies were the Environmental Protection Agency; the National Science Foundation; the Smithsonian Environmental Research Center; the Department of Agriculture; and the Atmospheric Environment Service, Canada. The spectral irradiances of participants’ calibrated standard lamps were measured at NIST prior to the Intercomparison. The spectral irradiance scales used by the participants agreed with the NIST scale within the combined uncertainties, and for all lamps the spectral irradiance in the horizontal position was lower than that in the vertical position. Instruments were characterized for wavelength uncertainty, bandwidth, stray-light rejection, and spectral irradiance responsivity, the latter with NIST standard lamps operating in specially designed field calibration units. The spectral irradiance responsivity demonstrated instabilities for some instruments. Synchronized spectral scans of the solar irradiance were performed over several days. Using the spectral irradiance responsivities determined with the NIST standard lamps, the measured solar irradiances had some unexplained systematic differences between instruments.

## 1. Introduction

Networks of spectroradiometers for monitoring solar ultraviolet irradiance at the surface of the Earth have been established by several government agencies in North America in response to concern over stratospheric ozone depletion. Detecting long-term trends in solar ultraviolet irradiance requires accurate measurements of the absolute irradiance for individual instruments, for the entire network, and between networks [1].

To assess the ability of spectroradiometers to accurately measure solar ultraviolet irradiance, and to compare these results between instruments of different monitoring networks, North American Interagency Intercomparisons of Ultraviolet Monitoring Spectroradiometers have been performed outside Boulder, Colorado. The first two such Intercomparisons were held September 19–29, 1994 and June 12–23, 1995. The experimental details and results from these efforts are described in [2, 3]. Results from the third Intercomparison, held June 17–25, 1996, are presented here. This Intercomparison was coordinated by the Optical Technology Division of the National Institute of Standards and Technology (NIST) and the Surface Radiation Research Branch (SRRB) of the National Oceanic and Atmospheric Administration (NOAA). Spectroradiometers from monitoring networks administered by the following agencies participated: the Environmental Protection Agency (EPA), the National Science Foundation (NSF), the Department of Agriculture (USDA), the Smithsonian Environmental Research Center of the Smithsonian Institution (SERC), and the Atmospheric Environment Service (AES) of Canada. A list of attendees is given in Appendix A.

The primary goal of the third Intercomparison was to improve upon the successes achieved at the previous two in the areas of instrument characterizations and synchronized solar irradiance scans. The instrument parameters of wavelength uncertainty, stray-light rejection, slit-scattering function, and spectral irradiance responsivity were characterized at all three Intercomparisons. All characterizations were done indoors at the first Intercomparison, while only the last was also performed outdoors. At the second Intercomparison, all the characterizations were done both indoors and outdoors, and the technique for measuring spectral irradiance responsivity was dramatically improved by using a field calibration unit. For this Intercomparison, all the characterizations were performed outdoors, and two field calibration units and power supplies were used for the spectral irradiance responsivity measurements. Comparisons between the different field calibration units and power supplies were performed on the first day of the Intercomparison and are described in [4]. There were no detectable differences between them. In addition, the spectral irradiances of participants’ standard lamps were measured at NIST prior to the Intercomparison to assess the spectral irradiance scales used by the participants.

While the participating networks remained the same for all three Intercomparisons, there were several new instruments at this Intercomparison. The AES instrument had a double monochromator, instead of a single monochromator as at the previous Intercomparisons, and the USDA instruments were new rotating shadowband radiometers. Unfortunately, the temperature controller of the NSF instrument broke at the beginning of the Intercomparison. Therefore, no results from this instrument are presented, despite the efforts by the participants to repair the instrument. Other instruments determined the atmospheric conditions during the Intercomparison, which will be useful for correlating these conditions with the measured solar ultraviolet irradiance. A list of all the instruments^1^ participating in the Intercomparison is given in Table 1.1.

The spectral irradiance responsivity measurements both checked the absolute irradiance scales used by the networks and provided a common scale for the synchronized measurements of solar irradiance. As at the previous Intercomparisons, these synchronized measurements were the most important aspect of this Intercomparison because they assess the present limits to which irradiances determined by different instruments can be compared. Note that all times given in this paper are in Universal Coordinated Time (UTC), which was 6 h ahead of Mountain Daylight Time, the local time.

## 2. Site Description

The site of the Intercomparison was Table Mountain, a plateau owned by the Federal government approximately 12.9 km north of Boulder, Colorado and 5.6 km east of the front range of the Rocky Mountains. This site was chosen because of its good view to the horizon, the presence of laboratory facilities, and the proximity of facility and staff support at both NIST and NOAA in Boulder.

For the synchronized measurements of solar irradiance, the spectroradiometers were located on individual concrete pads on the south side of the plateau at latitude 40.125° N, longitude 105.237° W, and elevation 1689 m. The pads were arranged in an east-west line and were 2.4 m square with 12.2 m between centers. The highest, and only major, obstruction to the horizon was a peak 5.6 km due west of the pads with a 5.1° angle of inclination. Temporary trailers approximately 30 m south of the pads housed the data acquisition and control computers and equipment for the spectroradiometers. The plateau sloped downward south of the pads, so the tops of the trailers were below the elevation of the pads. A test facility platform approximately 30 m west of the west-most pad is NOAA’s SRRB site. At the Intercomparison, pyranometers, pyrgeometers, radiometers, and shadowband radiometers were located on the platform. A meteorological tower recording the temperature, relative humidity, atmospheric pressure, and wind speed and direction at the site was located approximately 90 m northwest of the pads. Finally, a concrete building immediately to the southwest of the platform was used for servicing the instruments and holding meetings. A dome at the western end of the building was covered with a black cloth to eliminate reflections from it to the instruments.

## 3. Instrument Descriptions

Five instruments participated at the Intercomparison. A Brewer Spectrophotometer, Model MKIII, serial number 085, was operated by the participants from AES Canada. The instrument from the EPA network was also a Brewer Spectrophotometer, Model MKIV, serial number 101, and was operated by participants from the University of Georgia, who manage the EPA network. Participants from SERC operated a Smithsonian SR-18 Ultraviolet Scanning Radiometer, serial number UI. The instruments from the USDA network were Yankee Ultraviolet Rotating Shadowband Radiometers, serial numbers 270 and 271, and were operated by participants from Yankee Environmental Systems, Inc. (YES) and Colorado State University. For the remainder of this paper, these instruments will be designated AES, EPA, SERC, USDA 270, and USDA 271, respectively. The AES and EPA spectroradiometers operate by scanning a specified wavelength range. The signal is measured at discrete wavelengths within this range, from shortest to longest separated by a fixed interval. The SERC and USDA spectroradiometers operate at fixed wavelengths determined by filters in front of the detectors. Table 3.1 lists the characteristics of each instrument, and brief descriptions are given below.

### 3.1 Brewer Spectrophotometers

The Brewer Spectrophotometers measure total solar ultraviolet irradiance from 286.5 nm to 363 nm and total column O_3_, SO_2_, and NO_2_ from both direct sun and zenith sky measurements at specific ultraviolet wavelengths. A right-angle prism directs light from one of several sources—either internal calibration lamps, the sky, or a Teflon diffuser—along the optical path. This path contains apertures, filters, and lenses which focus the light onto the entrance slit of a single-grating (Model MKIV) or double-grating (Model MKIII) modified Ebert-type monochromator.

The exit slit focal plane of the monochromator contains six slits, five for selecting the wavelengths for determining the total column O_3_ and SO_2_ and one for wavelength calibration. A slotted cylindrical slitmask in front of the exit slit plane serves as the wavelength selector. The nominal bandwidth, set by the exit slits, is 0.6 nm. For a Model MKIV, the diffraction grating operates in third order and the first slit is selected for wavelengths shorter than 325 nm, and the grating is operated in second order and a different slit is used for longer wavelengths. The Model MKIII operates in third order over the entire wavelength range.

Light from the exit slit passes through a lens and a filter before focusing onto the cathode of a photomultiplier tube (PMT). The electrical pulses, generated by photons, from the PMT are amplified, discriminated, and divided by four before being transmitted to the counter. The MKIV model has an NiSO_4_ filter sandwiched between two Schott UG-11 filters for wavelengths shorter than 325 nm, and a single UG-11 filter for longer wavelengths, while the MKIII model has no filters in front of the PMT.

The wavelength of the monochromator in terms of micrometer steps was determined at the factory from the wavelengths of Hg emission lines. The wavelength registration of the monochromator is periodically checked and adjusted throughout a day by scanning the micrometer forward and backward about the 302.3 nm line from the internal Hg calibration lamp.

The two networks, AES and EPA, use different procedures for determining the spectral irradiance responsivity of their instrument from their spectral irradiance scale. The AES uses 1000 W DXW-type quartz-halogen lamps operating in the horizontal position 40 cm above the diffuser. The lamp is housed in a custom enclosure with air drawn over the lamp, and baffling limits the light falling on the diffuser to the direct beam from the lamp. The current from a power supply is monitored through a calibrated shunt resistor by a voltmeter so that the operator can manually adjust the current as needed. The EPA uses the set of calibration lamps, housing, and power supply furnished by the manufacturer. These are 50 W quartz-halogen lamps mounted horizontally 5 cm above the diffuser in a housing and operated at a constant 12 V.

### 3.2 Smithsonian Ultraviolet Scanning Radiometer

The Smithsonian SR-18 Ultraviolet Scanning Radiometer measures total solar ultraviolet irradiance at fixed wavelengths selected by 18 interference filters from 290 nm to 324 nm with nominal 2 nm bandwidths. The nominal and actual filter center wavelengths, bandwidths, and maximum filter transmittances of unit UI are given in Table 3.2. The filters are located on a filter wheel, which has a rotational frequency of 15/min underneath a Teflon diffuser. Light from the diffuser passes through each filter in turn, then through a three-aperture collimating apparatus, and is detected by a solar-blind PMT operating in current mode at 23 °C. The output current is converted to voltage and averaged for one minute for each filter. The spectral irradiance responsivity is determined at SERC by operating a calibrated 1000 W FEL-type quartz-halogen lamp in the horizontal position centered 50 cm above the diffuser.

### 3.3 Ultraviolet Rotating Shadowband Radiometer

The Ultraviolet Rotating Shadowband Radiometer (UVRSR) uses independent interference filter—photodiode detectors and an automated rotating shadowband to measure the direct-normal, total-horizontal, and diffuse-horizontal ultraviolet solar irradiance at seven wavelengths. The instrument is manufactured by YES following a similar design developed at the Atmospheric Science Research Center (ASRC) at SUNY, Albany. Two of these instruments, units 270 and 271, were at the Intercomparison. The instrument consists of two basic components: a detector assembly and an electronics enclosure. The detector assembly has a sensor head and the stepper motor-driven rotating shadowband, both mounted on a common base. The electronics enclosure contains the microprocessor and data acquisition and logging circuitry.

The diffuser used to collect the incident radiant flux and the detectors that measure it are located in the sensor head of the detector assembly. The diffuser is a Spectralon integrating cavity with a thin-walled top protruding above the top of the head and surrounded by a raised blocking ring. Two diaphragms of frosted WG-280 glass in the integrating cavity act as transmission diffusers. The geometry of the protruding diffuser, the blocking ring, and the integrating cavity was arrived at by extensive empirical optimization. Light exiting the bottom of the diffuser is incident on a hexagonal array of seven photodiodes with interference filters. The nominal and actual filter center wavelengths, and bandwidths of both units are given in Table 3.3. The output current of each photodiode is converted to a voltage using a separate transimpedance amplifier. The interior of the head is thermally insulated and has a thermostatic electrical heater that holds the temperature at 35 °C.

The shadowband is a strip of black metal formed into a circular arc with the face of the diffuser at the center of the arc. It is rotated around the polar axis by a stepping motor controlled by the microprocessor. The angle of the motor is adjusted for the latitude, and the azimuth is aligned with the Earth’s pole.

The microprocessor controls the operation of the instrument. At each measurement interval it computes the solar position using an approximation for the solar ephemeris. The measurement sequence starts with a measurement of the total-horizontal irradiance made while the band is below the head. The band is then rotated to make three measurements; the middle one blocks the sun and the other two block strips of sky to either side. These side measurements permit a first-order correction for the sky blocked by the band when the sun-blocking measurement is made. The average of these two side measurements is subtracted from the total-horizontal measurement and this correction is added to the sun-blocked measurement to determine the diffuse-horizontal irradiance. Finally the diffuse component of the irradiance is subtracted from the total-horizontal to produce the direct-horizontal component. Division by the cosine of the solar zenith angle then produces the direct-beam irradiance on a normal surface. The entire measurement sequence occurs four times per minute.

The control circuitry accumulates the data from the shadowband measurements. The signals from the detectors are amplified and multiplexed to an analog-to-digital converter with 12 bit plus sign resolution over the range −4.096 V to 4.095 V. The instrument can average over selected time intervals, one minute was used for the Intercomparison. The instrument stores the data using on-board memory and telemeters it with either an RS-232 or modem connection.

The spectral irradiance responsivities of both units were determined at both ASRC and YES prior to the Intercomparison using calibrated 1000 W FEL-type quartz-tungsten-halogen lamps. With the lamp illuminating the diffuser, the voltage from the photodiodes after amplification but before multiplexing were measured with a digital voltmeter.

## 4. Atmospheric Conditions

Weather conditions for the Intercomparison were marginally favorable. Prior to the synchronized solar scans, the skies were mostly clear with some afternoon clouds. However, atmospheric conditions for intermittently unsettled weather—a low pressure system along the West Coast and a high pressure system in the Midwest—also developed at this time. This unsettled weather occurred during the first three days of synchronized scans. Increasing cloudiness and strong winds on day 172 (sequential day of the year) were followed by cloudy skies on days 173 and 174, as well as heavy thunderstorms in the late afternoon and evening of day 173. A change in atmospheric conditions due to a low pressure system in the northeastern Pacific resulted in mostly clear skies on day 175, the last day of synchronized scans.

The temperature, relative humidity, barometric pressure, and wind speed and direction were recorded at the site of the Intercomparison by the instruments listed in Table 1.1. During the days of the synchronized scans (days 172 to 175), the temperature ranged from 15 °C in the early morning to nearly 30 °C in the late afternoon. The relative humidity varied from 40 % to 60 % during the synchronized scans, while the barometric pressure remained at approximately 83 kPa each day during the synchronized scans.

**Fig. 4.1:**
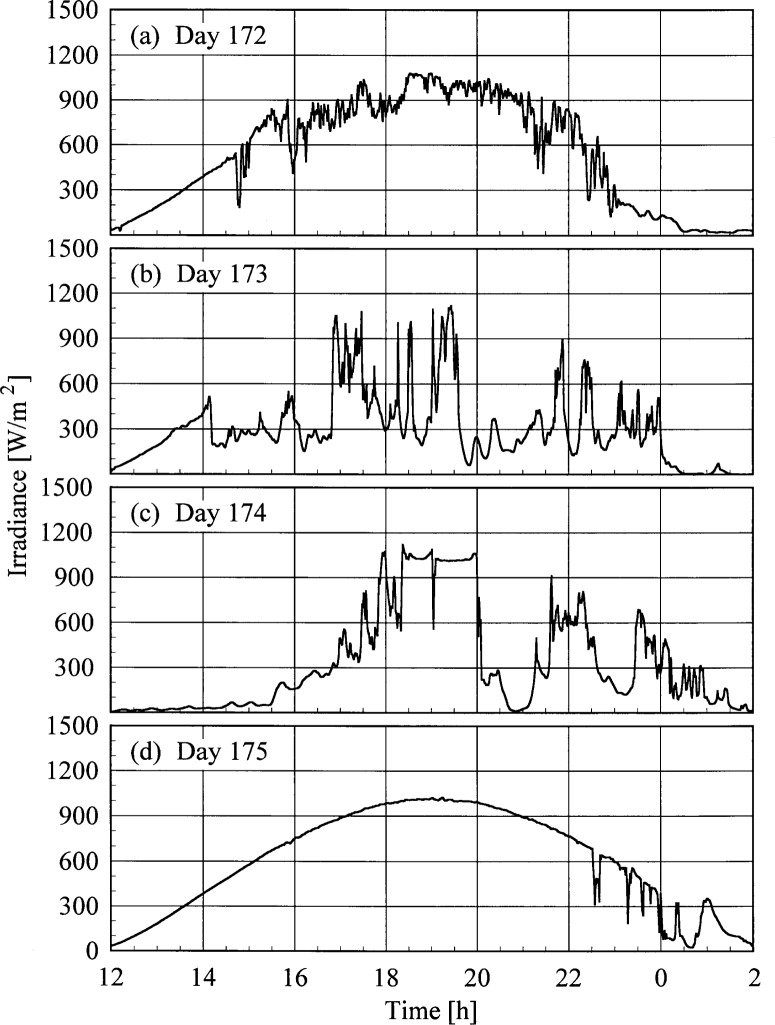
Total horizontal irradiance as a function of time from a solar pyranometer on the days indicated in the panels. Solar noon occurs at approximately 19.0 h UTC.

A set of broadband radiometric instruments, listed in Table 1.1, were located on the test facility platform and made continuous measurements concurrently with the Intercomparison. Results from one solar pyranometer are shown in Fig. 4.1, where the irradiance is plotted as a function of time for each day. This solar pyranometer measured total horizontal irradiance from 280 nm to 3000 nm. The cloudy conditions on day 173 and 174 are evident in Fig. 4.1, as are the partly cloudy conditions on day 172 and the clear skies during most of day 175.

**Fig. 4.2:**
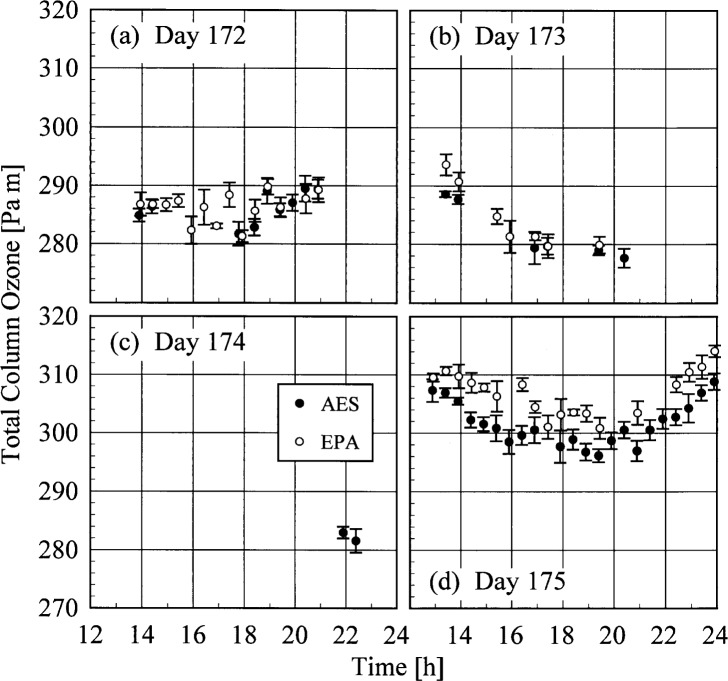
Total column ozone as a function of time on the days indicated in the panels as determined by the instruments indicated in the legend. The vertical bars are the standard deviations of the values.

The AES and EPA instruments determined total column ozone throughout the Intercomparison from measurements of the direct solar beam. The results are shown in Fig. 4.2, where the total column ozone is plotted as a function of time for each day. The vertical bars are the standard deviation of each value. The total column ozone was approximately 284 Pa m (280 matm cm) on days 172 and 173, while the sky was too cloudy on day 174 for accurate measurements. The total column ozone increased to between 299 Pa m and 309 Pa m (295 matm cm and 305 matm cm) on day 175, with a minimum occurring near solar noon.

## 5. Instrument Characterizations

The spectroradiometers were characterized for the parameters which most affect their ability to accurately measure solar ultraviolet irradiance, and which did not require elaborate experimental equipment or techniques. Therefore, the slit-scattering function, stray-light rejection, wavelength uncertainty, bandwidth, and spectral irradiance responsivity were determined. All of the characterizations were performed outdoors on the pads using techniques developed at the previous Intercomparisons. Because detailed mathematical discussions of the characterization techniques based upon a simple measurement equation have been given previously [2], they will not be repeated here.

### 5.1 Slit-Scattering Function and Stray-Light Rejection

#### 5.1.1 Experimental Procedure

An Omnichrome Model 3056 HeCd laser with a single line at 325.029 nm and a nominal power of 5 mW was used to determine both the slit-scattering function and the stray-light rejection of the instruments. The laser was mounted on a tripod, and a box with a hole in its side was placed on top of the instrument. The output of the laser was directed through the hole directly onto the diffuser. The background signal from the sky was minimized both by using the box and by performing the measurements at twilight and in the evening.

High-resolution spectral scans were performed near 325 nm to obtain the bandwidth of the instrument, the centroid of the line, and the shape of the slit-scattering function near its peak. Low-resolution spectral scans were performed across the entire wavelength ranges of the instruments to obtain the full slit-scattering function. For the SERC and USDA instruments, the signals were measured for 5 min. The AES and EPA instruments were configured so that the maximum signal did not saturate the PMT. This involved using an internal neutral-density filter for the high-resolution scans, and then removing the filter from the optical path for the low-resolution scans. A low-resolution scan was also performed with the laser beam blocked to check for stray light from sources other than the laser. There were no signals greater than the dark signal for any of the instruments.

#### 5.1.2 Data Analysis

While not important for spectral scans of laser lines because the light is monochromatic, background subtraction is important for spectral scans of lamp emission lines because of the underlying continuous emission from these lamps. To maintain consistency, background subtraction was also performed for spectral scans of laser light. The background signal is described by a linear fit of the signals at wavelengths that differ by 1.5 bandwidths from the wavelength of the peak signal. For unresolved multiple lines in emission lamps, the factor is increased from 1.5 to 2.0. The signals and wavelengths for the first 5 consecutive data pairs that lie outside this range are averaged and fit with a straight line to yield the background signal as a function of wavelength. This fit is subtracted from the signals within the range. There is obviously an interplay between the background subtraction and the bandwidth, but a consistent bandwidth can be obtained after only one or, at most, two iterations between background subtraction and the bandwidth calculation.

The bandwidth of the instrument is defined here as the full-width-at-half-maximum (FWHM) from a high-resolution spectral scan of a laser line or a singlet lamp emission line. Linear interpolation is used to find the wavelengths at which the signal is one-half that of the peak. The bandwidth is then the difference between these two wavelengths.

The centroid method is used as the best estimate of the wavelengths of laser lines and lamp emission lines. The centroid *C* from a high-resolution scan is given by

$$C=\sum_{i}S_{i}\lambda_{i}/\sum_{i}S_{i},$$ (5.1)

where *i* indexes the signals *S* and wavelengths λ of those signals greater than 0.1 times the peak signal.

Because the signal from the laser saturated without a neutral-density filter in the optical path of the AES and EPA instruments, the optical densities of the filters at 325 nm were determined from the common wavelengths at which signals were measured for scans both with and without the filters. For the high-resolution scans, normalization of the signals by the peak signals was straight-forward because there was no saturation. For the low-resolution scans, the peak signals from the high-resolution scans and the optical densities of the filters were used to calculate the peak signals for the scans without the neutral-density filters.

The peak signal for the SERC instrument was not readily known because there is no filter centered at 325 nm. Therefore, the peak signal for each filter was obtained from the measured signal of the filter centered at the longest wavelength that did not saturate. These peak signals were calculated by dividing the measured signal from the filter centered at 320.28 nm by the transmittance of that filter at 325 nm and multiplying by the peak transmittance of each filter. A similar analysis technique was attempted for the USDA instruments, but was not successful because there was insufficient dynamic range for the filter transmittances at 325 nm.

#### 5.1.3 Results and Discussion

The bandwidths of the instruments and the centroids of the laser line are most useful when included with those values obtained from the scans of the Cd, Hg, and Zn lamps. Therefore, the results from these determinations are shown in Figs. 5.3 and 5.4. The bandwidths at 325 nm are close to the nominal value of 0.6 nm for the AES and EPA instruments.

**Fig. 5.1:**
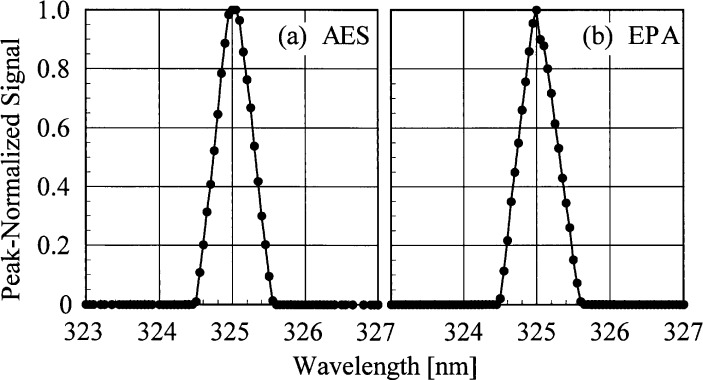
Peak-normalized signal as a function of wavelength from high-resolution spectral scans of the 325.029 nm line from a HeCd laser for the instruments indicated in each panel, demonstrating the slit-scattering functions.

**Fig. 5.2:**
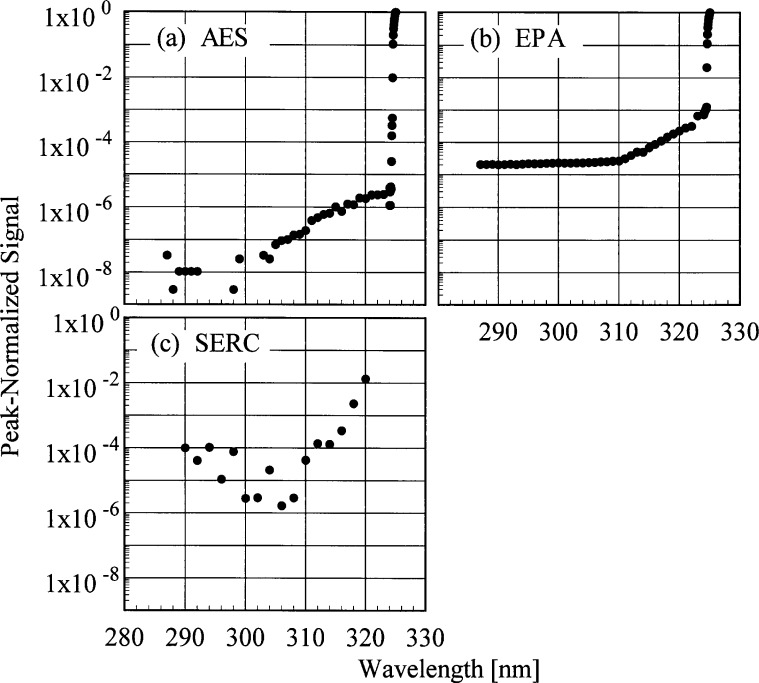
Peak-normalized signal as a function of wavelength from low-resolution spectral scans of the 325.029 nm line from a HeCd laser for the instruments indicated in each panel, demonstrating the stray-light rejections.

The slit-scattering functions are shown in Figs. 5.1 and 5.2, from high- and low-resolution scans, respectively, where the peak-normalized signal is plotted as a function of wavelength. From Fig. 5.1, the slit-scattering functions of the AES and EPA instruments are nearly triangular and symmetric about the peak wavelength. The stray-light rejection of each instrument, from Fig. 5.2, is the peak-normalized signal at the shortest wavelengths. The stray-light rejections of approximately 10^−8^ and 10^−5^ are reasonable for the AES and EPA instruments because they are double- and single-grating instruments, respectively. The stray-light rejection of the SERC instrument, approximately 10^−5^, is also reasonable for interference-type filters.

### 5.2 Bandwidth and Wavelength Uncertainty

#### 5.2.1 Introduction

Characterizing the instruments in terms of their response to light from Cd, Hg, and Zn emission line lamps is somewhat more complex than was the case for a HeCd laser both because there is a continuum in addition to the lines and because there can be unresolved multiple lines. However, it is useful because it yields information at several wavelengths about the bandwidth and the wavelength repeatability and uncertainty of the instruments. The wavelength uncertainty is especially important in the UV-B region of the solar spectrum (280 nm to 315 nm) because the irradiance at the Earth’s surface changes rapidly with wavelength, so a small uncertainty in wavelength translates into a large uncertainty in irradiance.

A distinction needs to be made between wavelength calibration and wavelength registration, both of which affect the wavelength uncertainty. The wavelength calibration is the relation between the motor steps that determine the grating angle and the monochromator wavelength, and is determined from the emission lines of a Hg lamp. The wavelength calibration is in general a non-linear function of motor steps. Therefore, the lines from the Cd and Zn lamps are especially valuable for determining the wavelength uncertainty because these lines are not used in the original calibrations of the instruments. The wavelength registration is a fixed offset of motor steps from a known position, and is provided by the 302.3 nm line of Hg for the AES and EPA instruments.

The wavelengths of emission lines from gas lamps are known to a high degree of accuracy; however, the relative intensities of these lines change with lamp and operating condition. Therefore, an Oriel Model 6035 Hg emission lamp was used because of recent measurements of the relative intensities of the lines from this particular model of lamp [5, 6]. The Cd and Zn lamps were purchased from BHK, Inc.

#### 5.2.2 Experimental Procedure

The Cd, Hg, and Zn emission lamps were mounted horizontally in separate aluminum enclosures that fit over the diffusers and reduced the background light, especially from the sky. The lamps were warmed up for 10 min and a spectral scan was performed by the instrument. The AES and EPA instruments performed spectral scans over their entire operating ranges at 0.05 nm increments.

#### 5.2.3 Data Analysis

Background subtraction and calculation of the centroid and bandwidth for each line were performed as detailed in Sec. 5.1.2. Only the bandwidths for single lines were taken to be indicative of the bandwidth of the instrument at that wavelength. The actual centroids of the lines were calculated from the wavelengths and relative intensities of the emission lines for that particular model of Hg lamp and from the published values for Cd and Zn emission lines [7].

#### 5.2.4 Results and Discussion

**Fig. 5.3:**
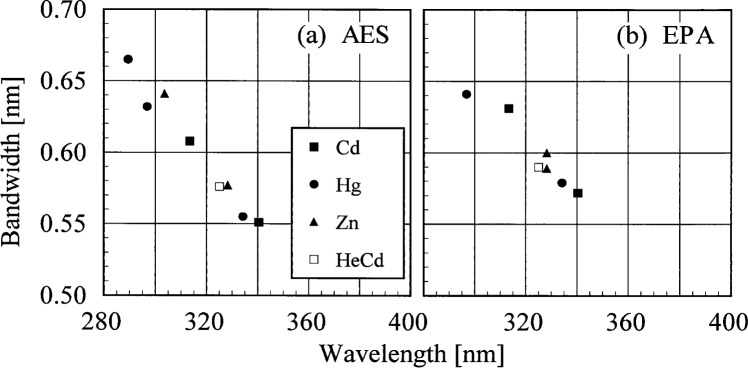
Bandwidth as a function of wavelength for the instruments indicated in each panel from high-resolution spectral scans of the singlet lines from the sources indicated in the legend.

**Fig. 5.4:**
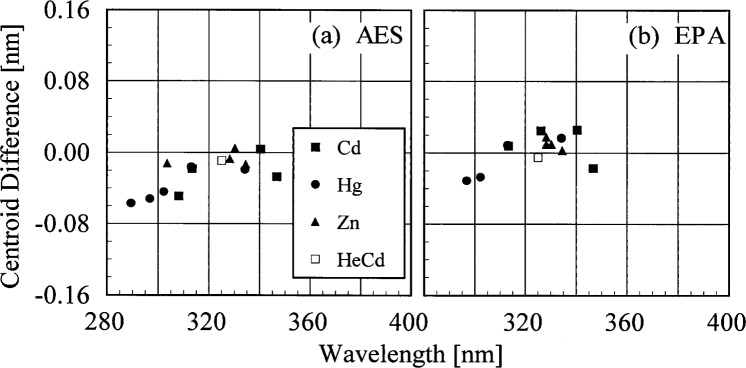
Centroid difference between the calculated and actual values for the instruments indicated in each panel from high-resolution spectral scans of the lines from the sources indicated in the legend, demonstrating the wavelength uncertainty of each instrument.

The bandwidths calculated from the measurements of singlet Cd, Hg, and Zn lines and the HeCd line are plotted in Fig. 5.3 as a function of wavelength. Likewise, the differences between the calculated and actual centroids of the Cd, Hg, Zn, and HeCd lines are plotted in Fig. 5.4 as a function of wavelength.

The bandwidths of the AES and EPA instruments decrease with increasing wavelength, and this decrease is consistent between sources and instruments. These results are similar to those obtained at the prior Intercomparisons [2, 3]. There is a systematic trend for the centroid differences of the AES and EPA instruments which is consistent between sources. The wavelength uncertainty varies between −0.06 nm and 0 nm for the AES instrument, and between −0.04 nm and 0.04 nm for the EPA instrument.

### 5.3 Spectral Irradiance of Standard Lamps

#### 5.3.1 Introduction

Both the 1994 and 1995 Intercomparisons showed that the participants’ spectral irradiance scales differed from the NIST scale by as much as 10 %. As a first step in trying to determine the cause of this discrepancy, the spectral irradiances of calibrated lamps, one from each participant except USDA, were measured at NIST prior to the 1996 Intercomparison. These measurements were performed in conjunction with calibrations of NIST standard lamps, and were done for both vertical and horizontal lamp orientations.

The experimental technique used to measure the spectral irradiance of standard lamps was the same as that used previously [8], except for a different spectroradiometer. The key to the experiment was an integrating sphere that rotated to view either a vertical or a horizontal lamp.

Lamps from both NIST and EPA were 1000 W modified FEL-type, while the lamp from SERC was also a 1000 W FEL-type but without the modified bipost base. The lamps from AES and NSF were 1000 W and 200 W DXW-type, respectively.

The spectral irradiance standards for the experiment were three secondary standard lamps, designated E-003, E-006, and E-009, calibrated in the vertical position using three primary standard lamps, designated F-305, F-315, and F-410, calibrated by NIST. The horizontal standard lamps for NIST, designated E-002, E-004, E-007, and F-332, were calibrated in the horizontal and vertical positions. The EPA lamp GS-919 had been calibrated at the University of Georgia in both the vertical and horizontal positions using standard lamps calibrated at NIST. The SERC lamp, EN-74, and the AES lamp, S-849, had been calibrated in the vertical position by Eppley Laboratories and Optronic Laboratories, respectively. The NSF lamp M-881 was calibrated in the horizontal position by Optronic Laboratories and also using standard lamps at Biospherical Instruments, Inc.

#### 5.3.2 Experimental Procedure

The mounts for the lamps were attached to tilt and translation stages so they could be properly aligned. For the vertical lamps, the stages were attached to an optical table, while they were attached to a vertical aluminum rod on the table for the horizontal lamps. The optic axes for both lamp orientations were determined using HeNe lasers behind or above the lamp mounts. A glass slide was placed over the entrance port of the integrating sphere and the retroreflected beam from the center of the port defined the optic axis.

The mount for the modified FEL-type lamps was aligned as in [8] using a lamp jig. The jig was placed in the mount and aligned to be centered on and perpendicular to the optic axis and 50.0 cm from the entrance port. Because the FEL-type lamp from SERC did not have a modified base, the mount for the lamp supplied by SERC was attached directly to the tilt stage. The mount was leveled so that its front plane was perpendicular to the optic axis, and the filament of the lamp was centered on the optic axis and 50.0 cm from the entrance port.

For the 1000 W DXW-type AES lamp the tilt stage was removed and the mount was attached directly to the translation stage. The filament was centered on the optic axis at a distance of 50.0 cm from the entrance port to the center of the filament. The lamp was rotated so that the flat faces of the tabs at the ends of the lamp were perpendicular to the optic axis, and the nipple on the lamp envelope was on the left side when looking along the optic axis toward the integrating sphere. The 200 W DXW-type NSF lamp was permanently attached to its mount. The mount was placed on the translation stage so that the lamp was horizontal and the nipple on the lamp envelope was on top. The mount was leveled and translated so that the nipple was on the optic axis and the center of the filament was 50.0 cm from the entrance port.

The spectroradiometer used for the experiments consisted of an integrating sphere, imaging optics, monochromator, and detector. The integrating sphere had an interior diameter of 5 cm and was coated with PTFE powder. The 1 cm^2^ entrance port and 0.5 cm^2^ exit port were 90° from each other. The sphere was mounted on rotation and translation stages and aligned so that the exit port and rotation axis were centered on the optic axis of the imaging system. The imaging system had plane and spherical mirrors that imaged the exit port of the integrating sphere onto a mask with a 2 mm by 2 mm hole in front of the entrance slit of the monochromator. The alignment of the integrating sphere was checked to ensure that the same area of the exit port was imaged onto the hole in both sphere orientations.

The prism-grating 2/3 m monochromator had a 600 groove/mm grating blazed at 300 nm and was set for a 1 nm bandpass. The wavelength was calibrated by illuminating the entrance port of the integrating sphere with a Hg emission lamp. Signals were measured as a function of the encoder attached to the monochromator wavelength drive for Hg lines from 253.6 nm to 435.8 nm. The centroids of the lines in terms of encoder units were fit to the actual line wavelengths with a second-order polynomial with an uncertainty of 0.05 nm. The detector was a cooled bi-alkali photomultiplier tube operating in the photon counting mode. The dark signal count was less than 10/s.

The lamps were operated with a DC current from a power supply, which was controlled using a computerized feedback circuit described in [9]. The NIST lamps were operated at 8.2 A, the AES and EPA lamps at 8.0 A, the SERC lamp at 7.9 A, and the NSF lamp at 6.5 A. The voltages across the lamps were also monitored.

A single measurement of a lamp consisted of two wavelength scans from 250 nm to 400 nm every 10 nm. Photon counts with 2 s integration times were accumulated five times at each wavelength. The first scan was performed while the lamp was warming up. The direct beam from the lamp to the integrating sphere was blocked with a black cylinder to measure the diffuse signal. After the lamp had operated for at least 15 min, the second scan was performed with the cylinder removed to measure the total signal. The five readings were averaged and the diffuse signal was subtracted from the total signal to yield the direct signal.

#### 5.3.3 Data Analysis

The spectral irradiance responsivity *R*(*λ*) of the spectroradiometer was determined throughout a day’s measurements using the secondary standard lamps. Knowing the spectral irradiance *E*_s_(*λ*) of a standard lamp, *R*(*λ*) is given by

$$R(\lambda )=S_{s}(\lambda )/E_{s}(\lambda ),$$ (5.2)

where *S*_s_(*λ*) is the direct signal from the standard lamp. The signals from the NIST field standard lamps and the participants’ lamps were measured with the lamps in both the vertical and horizontal orientations. The single exception to this was the NSF lamp, which was measured in only the horizontal orientation. From the direct signal *S*_l_(*λ*) of a lamp, the spectral irradiance *E*_l_(*λ*) is given by

$$E_{1}(\lambda )=S_{1}(\lambda )/R(\lambda ).$$ (5.3)

The components of uncertainty that contribute to an uncertainty in the spectral irradiance of a lamp are the spectral irradiance of the standard lamp, the currents supplied to the lamps, the alignment of the lamps, the wavelength of the monochromator, the responsivity stability of the spectroradiometer, and the signals. The resulting uncertainties were evaluated using the technique detailed in Appendix D of [3].

#### 5.3.4 Results and Discussion

**Fig. 5.5:**
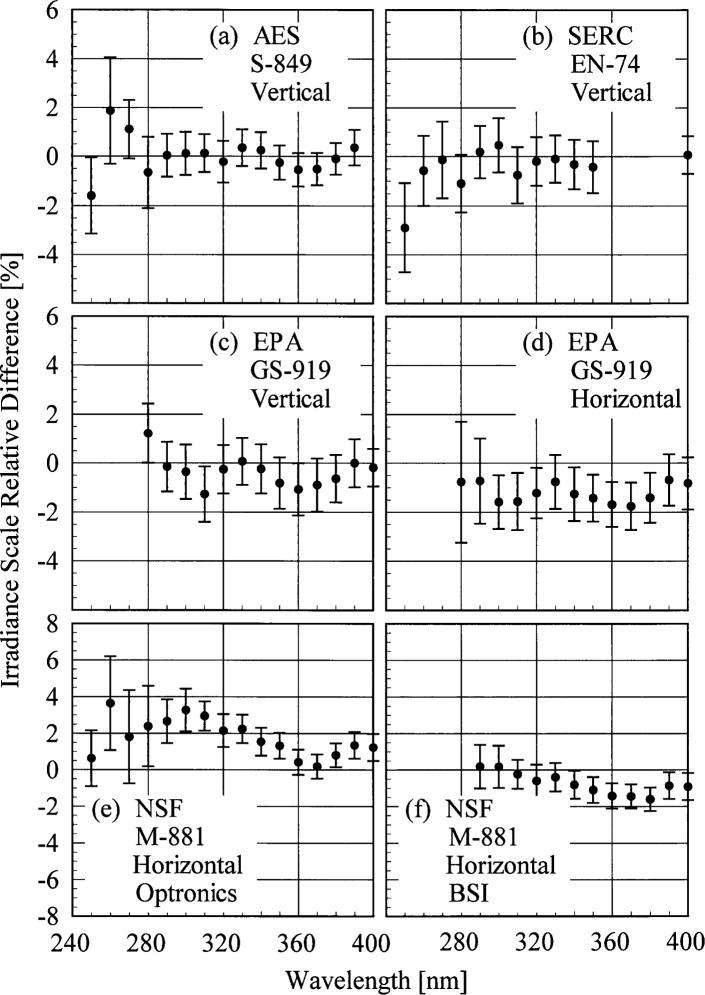
Relative difference between the participants’ spectral irradiance scales and the NIST spectral irradiance scale as a function of wavelength determined prior to the Intercomparison. The participants, lamps, and lamp orientations are indicated in each panel, the sources of the calibration are indicated in (e) and (f), and the vertical lines are the standard uncertainties.

The primary purpose of this experiment was to compare the scales of spectral irradiance of the participants to the NIST scale. The relative difference between the spectral irradiance assigned to a lamp by the participant and the spectral irradiance measured at NIST indicates the agreement between the scales. Note that the relative difference between two values *x* and *y* is given by (*x* − *y*)/*y* = *x*/*y* − 1. The relative difference as a function of wavelength is shown in Fig. 5.5 for each lamp, with the vertical bars indicating the combined uncertainty. The relative differences for all the vertical lamps are zero within the uncertainties of the measurements, as shown in Figs. 5.5 (a) to (c). The horizontal lamps, however, have finite relative differences. The relative differences of both the EPA lamp and the NSF lamp calibrated by BSI, shown in Figs. 5.5 (d) and (f), are approximately −1 %, suggesting that the NIST scale for horizontal lamps may be 1 % too high. The scale used by Optronics for horizontal lamps may also be incorrect because the relative differences shown in Fig. 5.5 (e) are not similar to those for the other horizontal lamps. Additional experiments are required to definitively determine the cause of the finite relative differences between the spectral irradiance scales for the horizontal lamps.

**Fig. 5.6:**
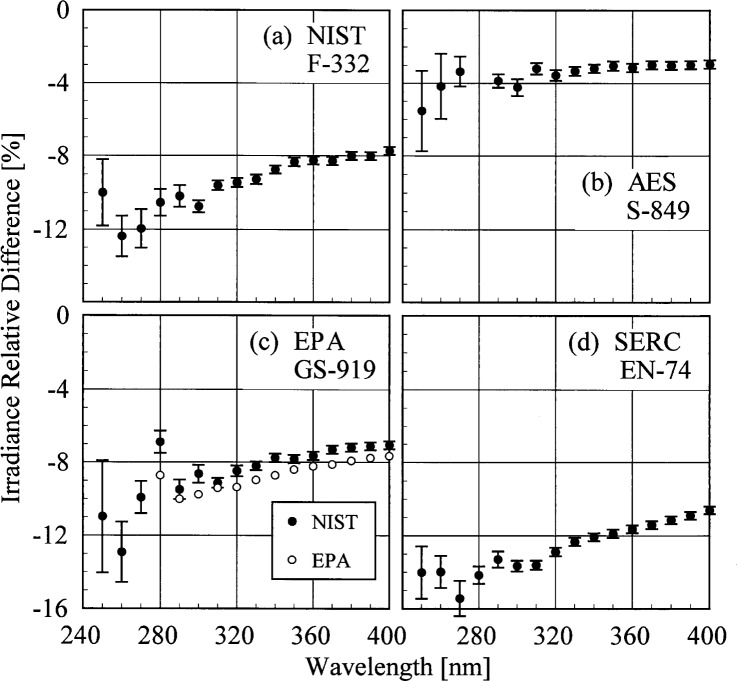
Relative difference between the irradiances in the horizontal lamp position and the vertical lamp position as a function of wavelength determined prior to the Intercomparison. The participants and lamps are indicated in each panel, the sources of the calibration are indicated in the legend in (c), and the vertical lines are the standard uncertainties.

A secondary purpose was to determine the effect on the spectral irradiance of operating a lamp in a horizontal position. The relative difference as a function of wavelength between the spectral irradiance of a lamp operated in a horizontal position to that of the lamp in the vertical position is shown in Fig. 5.6. The vertical bars indicate the combined uncertainty of the relative difference. The relative difference of the NIST lamp is consistent with results obtained previously and detailed in [8]. The spectral irradiance of the AES lamp is less affected by its orientation, while the relative difference for the EPA lamp, from both this experiment and the calibrations performed at the University of Georgia, are similar to that of the NIST lamp. Even though the SERC lamp, like the ones from NIST and EPA, is a 1000 W FEL-type, the spectral irradiance decreases much more in the horizontal position than it does for the other lamps.

These measurements were worthwhile for three reasons. First, measuring the participants’ lamps gave NIST valuable experience with the issues of aligning and operating these lamps. Second, with the exception of the NSF lamp, the spectral irradiance calibrations of the lamps agreed with the spectral irradiance measured using the NIST scale. On the one hand, this is an encouraging result because there is consistency between scales. On the other hand, it implies that the discrepancy between the NIST scale and the participants’ scales observed at the Intercomparisons is arising during the transfer of the scales from the standard lamps, such as those measured in this experiment, to the lamps used at the Intercomparisons. Third, the measurements of lamps in the horizontal position showed that the spectral irradiance of all the lamps decreases relative to its values when the lamp is vertical. Therefore, the lamps must be calibrated in the horizontal position if they are going to be used to accurately determine the responsivities of instruments in the field.

### 5.4 Spectral Irradiance Responsivity

#### 5.4.1 Introduction

Measuring the spectral irradiance responsivity (hereafter termed simply the responsivity) of the instruments, both with the NIST standard lamps and with the standard lamps of the participants, was the most important characterization performed at the Intercomparison. As at the other Intercomparisons, these measurements determined the agreement between the spectral irradiance scales, the temporal stability of the instruments, and the responsivity of each instrument for the synchronized solar ultraviolet irradiance measurements.

The responsivity of every instrument was determined outdoors on the pads. Two field calibration units were used for the measurements using the NIST standard lamps, one built by NIST and the other built by NOAA, as well as two power supplies designated the laboratory unit and the field unit. Consistency between the field calibration units, and the power supplies used to operate them, was established from measurements performed indoors on the first day of the Intercomparison and detailed in [4]. The participants’ standard lamps were also operated outdoors in their own enclosures.

#### 5.4.2 Experimental Procedure

Because the field calibration unit performed well at the previous Intercomparison, all the responsivities at this Intercomparison were measured outdoors. All the instruments were measured on three separate days using the NIST standard lamps operating in the field calibration units. The lamp mounts were aligned once to center the lamp 50.0 cm above the diffuser and checked on each instrument. The only problem with using the field calibration units was that the interface plate for the USDA instruments did not fit correctly, nor did the alignment tool. Therefore, the interface plate for the NIST unit was modified and the height of the unit was set using spacers of various thickness to achieve the correct distance as determined with the laboratory distance indicator. The spectral irradiance of the 1000 W FEL-type NIST standard lamps, designated E-002 and E-004, had been determined in the horizontal position as detailed in Sec. 5.3.

For all determinations of responsivity using the NIST standard lamps, spectral scans were performed with a circular shutter halfway between the lamp and the diffuser to measure the diffuse signal, and without the shutter to measure the total signal. For both Brewer instruments, the wavelength registration was set prior to measuring the responsivity. For the AES instrument, spectral scans were performed from 286.5 nm to 349.5 nm at 3.5 nm increments, one scan for the diffuse signal and two scans for the total signal. A similar procedure was used for the EPA instrument, except that the spectral scans were performed to 360 nm. Both the diffuse and total signals from the SERC and USDA instruments were collected for 10 min.

The participants with the AES instrument used 1000 W DXW-type lamps mounted 40 cm above the diffuser in a custom enclosure. The lamps were designated S-702, S-790, and S-849. The EPA instrument used 50 W quartz-tungsten-halogen lamps, mounted 5 cm above the diffuser in an enclosure and designated 271, 272, 273, 274, and 275. The lamps were supplied by Sci-Tec, Inc. and calibrated both by this company and by the University of Georgia. The responsivity of the SERC instrument had been determined at the home laboratory with a 1000 W FEL-type quartz-tungstenhalogen lamp, supplied and calibrated by Eppley Laboratories, designated EN-74. Likewise, the responsivities of the USDA instruments had been determined both at ASRC and at YES prior to the Intercomparison.

A schedule of the spectral scans of standard lamps is given in Table 5.1, along with the corresponding instrument temperatures. Standard lamp E-002 was operated in the NIST field calibration unit using the field power supply, while lamp E-004 was operated in the NOAA field calibration unit using the laboratory power supply. The AES instrument began the Intercomparison without a neutral-density filter in the optical path. However, the initial measurements of the solar irradiance indicated that such a filter was needed, so one was used for subsequent measurements. The first responsivity measured with lamp E-004, on day 171, was without the neutral-density filter, as were the second and fourth measurements using lamp S-790 on day 173. All the other measurements of responsivity for the AES instrument had the neutral-density filter in the optical path. A thunderstorm prevented a measurement of the responsivity of the USDA 270 instrument on day 173.

#### 5.4.3 Data Analysis

From spectral scans of a standard lamp, the responsivity is given by dividing the signal by the lamp irradiance. For the NIST standard lamps, the signal was the direct signal, given by the difference between the total signal and the diffuse signal. However, for the participants’ lamps, the signal was the total signal because a shutter was not used to measure the diffuse signal. The spectral irradiances of the standard lamps were fit with a cubic spline interpolation to the wavelengths of the signals. For all the instruments, the signals at each wavelength from multiple scans were averaged. The standard uncertainties in the signals were the standard deviations of the mean, and these were propagated through to the direct signals.

The uncertainty analysis for the responsivities is the same as that given in Appendix D of [3]. Components of uncertainty arise from the standard lamp (spectral irradiance, size of diffuser, goniometric distribution, and current), the alignment of the lamp, and the instrument (wavelength and signal). The relative standard uncertainties arising from each component are given in Table 5.2 at selected wavelengths for the second determination of responsivity with the field calibration unit. The relative standard uncertainties are combined in quadrature for both random and systematic effects. The greatest systematic component is the irradiance of the standard lamp, while the greatest random component is the signal. Note that these uncertainties apply only to the NIST standard lamps. For the participants’ lamps, only the uncertainties arising from the instrument (the wavelength and signal) are known.

The separation of uncertainties between random and systematic effects is important when comparing responsivities. For example, the relative standard uncertainty in the relative difference between the responsivities determined by a NIST standard lamp and by a participant’s lamp includes components of uncertainty arising from both random and systematic effects. However, the relative standard uncertainty in the relative difference between two responsivities determined by a NIST standard lamp includes components of uncertainty arising only from random effects.

#### 5.4.4 Results and Discussion

The spectral irradiance of each participant’s lamp is based upon the spectral irradiance scale used by that participant’s monitoring network. These scales, in turn, are based upon calibrated lamps supplied by different manufacturers. A comparison between these scales and the NIST spectral irradiance scale is very important to assess the accuracy of the participants’ scales. The relative difference between a participant’s spectral irradiance scale and the NIST scale is given by the relative difference between the responsivity using the NIST standard lamp and the responsivity using the participant’s standard lamp, assuming that the responsivity of the instrument remains stable over the time period between the two measurements.

**Fig. 5.7:**
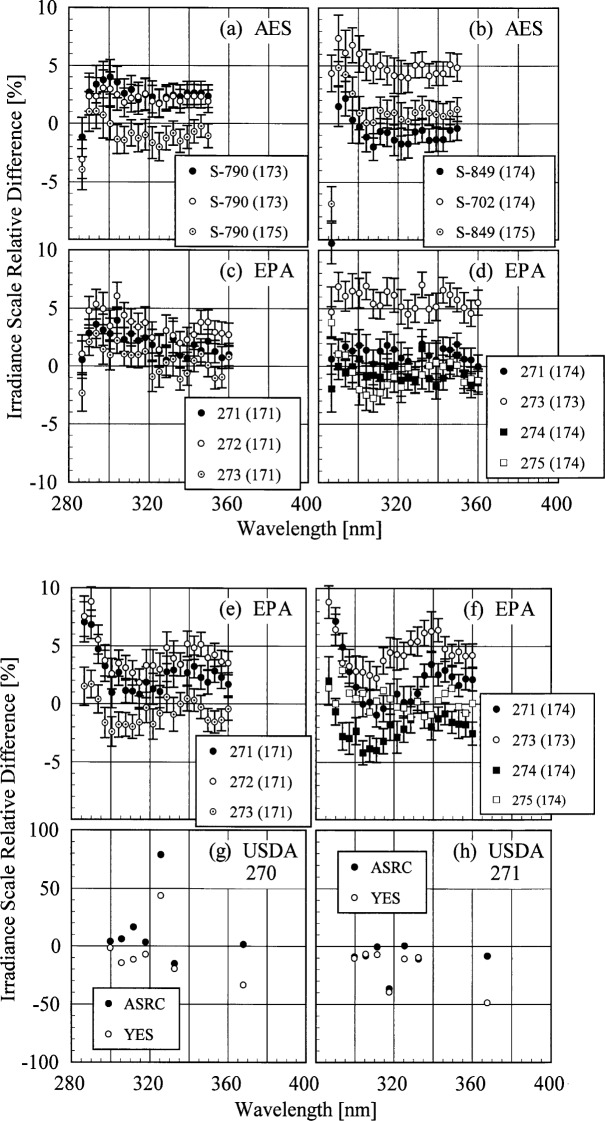
Relative difference between the participants’ spectral irradiance scales and the NIST spectral irradiance scale as a function of wavelength. The instruments are indicated in each panel, the participant’s lamps and the days on which they were measured are indicated in the legends, and the vertical lines are the standard uncertainties. In (c) and (d) the EPA scale was based upon calibrations performed at the University of Georgia, while in (e) and (f) the scale was based upon calibrations performed at Sci-Tec. The locations of the calibrations upon which the USDA scale was based are indicated in the legends in (g) and (h).

**Fig. 5.8:**
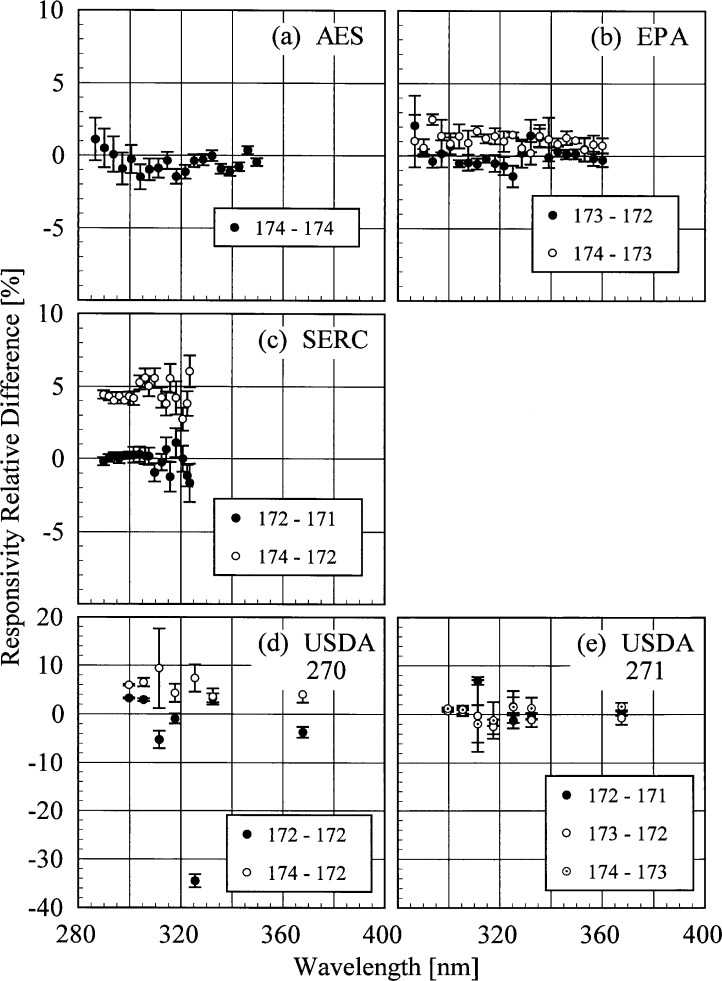
Relative difference between two responsivities determined using the NIST standard lamps as a function of wavelength, indicating the temporal stability of the instruments. The instruments are indicated in each panel, the days on which the responsivities were determined are indicated in the legends, and the vertical lines are the standard uncertainties.

**Fig. 5.9:**
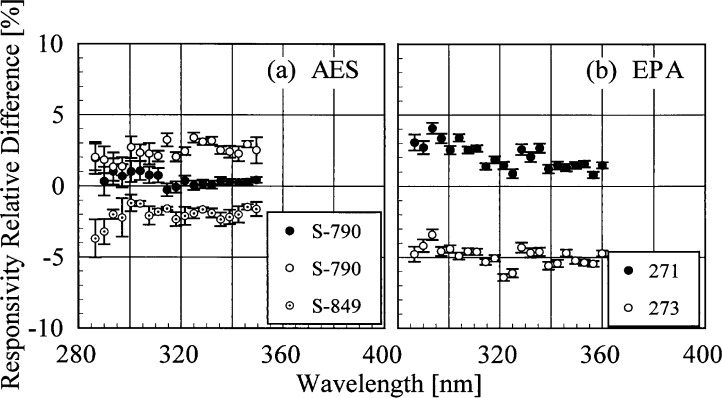
Relative difference between two responsivities determined using the participants’ lamps as a function of wavelength, indicating the temporal stability of the instruments. The instruments are indicated in each panel, the participants’ lamps are indicated in the legends, and the vertical lines are the standard uncertainties.

The participant responsivities used for this comparison were those that were determined on the same day as a scan of the NIST standard lamp, or from measurements performed prior to the Intercomparison. The relative difference between the participant’s spectral irradiance scale and the NIST scale as a function of wavelength is shown in Fig. 5.7. The vertical bars are the combined standard uncertainties of the differences using components arising from both random and systematic effects. The lamps, times, and instrument temperature changes used for the differences in these two figures, as well as in Figs. 5.8 and 5.9, are listed in Table 5.3.

The spectral irradiance scales from the lamps used by AES and EPA are generally within ± 5 % of the NIST scale. The AES scale from lamp S-790, shown in Fig. 5.7 (a), was systematically 2 % to 3 % greater than the NIST scale on day 173 and systematically 1 % to 2 % lower on day 175. From Fig. 5.7 (b) the relative difference between the scales using lamp S-849 was within ± 1 % on both days, while the scale using lamp S-702 was systematically 4 % to 5 % greater than the NIST scale. The relative difference for both of these lamps increased significantly at the shortest wavelengths. The EPA scale using the University of Georgia calibrations, shown in Figs. 5.7 (c) and (d), was within ± 5 % of the NIST scale for all the lamps except 273 on day 173, which was 5 % to 7 % greater. The scale from some lamps agreed with the NIST scale within the uncertainties, while the scales with the other lamps were systematically greater or less than the NIST scale. The relative differences obtained with the Sci-Tec calibrations, shown in Figs. 5.7 (e) and (f), were larger than those obtained with the University of Georgia calibrations. In addition, the relative differences increased at the shortest wavelengths.

The spectral irradiance scales used to calibrate the USDA instruments had large relative differences with the NIST scale, as shown in Figs. 5.7 (g) and (h). These differences ranged from −50 % to 80 %, with little consistency between the instruments, wavelengths, or the origin of the scale. The smallest relative differences were obtained for instrument 271 using the ASRC scale, although even that had a relative difference of −40 % at 317 nm.

The responsivity of every instrument was determined from two to four times using the NIST standard lamps. In addition, the responsivities of the AES and EPA instruments were measured using the participants’ lamps. The relative difference between responsivities determined with the same lamp at two different times indicates the temporal stability of the instrument. These relative differences as a function of wavelength are shown in Fig. 5.8 from spectral scans using the NIST standard lamps and in Fig. 5.9 using the participants’ lamps. The vertical bars are the combined standard uncertainties of the differences using components arising from only random effects.

From Fig. 5.8, the AES instrument was stable to within ± 2 % over the 18 h between spectral scans of the NIST standard lamp on day 174. The EPA instrument was also stable to within ± 2 %, but over a longer time of nearly 70 h from day 172 to day 174. The SERC instrument was stable to within ± 2 % from day 171 to day 172, a time of 24 h, but the responsivity increased by 3 % to 5 % from day 172 to day 174, a time of 43 h. This increase may be due to a decrease in the filter temperature of 14.6 °C between the two measurements of responsivity. The USDA instrument 270, however, was not as stable as the others. It was stable to within −6 % to 4 % over 22 h on day 172, except at 326 nm, where the responsivity decreased by 34 %, while it was stable to within 2 % to 10 % over 43 h from day 172 to day 174. Instrument 271 was stable to within ± 2 % over the 66 h from day 171 to day 174, except for one 7 % increase in responsivity at 300 nm.

The temporal stability of the AES and EPA instruments measured with the participants’ lamps, shown in Fig. 5.9, does not agree with the stability determined with the NIST standard lamps. Results obtained using lamp S-790 showed that the AES instrument was stable for a time of less than an hour, but that the responsivity increased by 1 % to 3 % over 24 h. Conversely, the responsivity from spectral scans of lamp S-849 decreased by 1 % to 4 % over 9 h. Because this instrument does not have a NiSO_4_ filter, temperature should not have a significant effect on responsivity, as it does in other Brewer instruments [2]. Similar inconsistencies were obtained for the EPA instrument. While lamp 271 showed that the responsivity increased by 1 % to 4 % over 69 h, the responsivity from lamp 273 decreased by 4 % to 6 % over 48 h. These changes in responsivity are not correlated with temperature differences, as was observed at the 1994 Intercomparison [2]. The temperature decreased between the measurements of responsivity for both lamps, while the responsivity decreased for one and increased for the other.

Several conclusions follow from the responsivities determined at the Intercomparison. While the spectral irradiance scales of AES and EPA measured at NIST prior to the Intercomparison agreed with the NIST scale, shown in Fig. 5.5, the scales disagreed by approximately 5 % at the Intercomparison. The most plausible explanation is a problem in the transfer of these scales from the primary lamps used by these participants to the lamps used at the Intercomparison. As for the USDA instruments, because the relative differences shown in Figs. 5.7 (g) and (h) are not consistent between the two instruments at the same wavelengths, the instruments are not stable upon movement. This conclusion is further supported by the inconsistency between the responsivity calibrations at ASRC and YES.

The responsivity of the SERC instrument was definitely not stable over several days, as shown in Fig. 5.8 (c), which might be due to a temperature effect. There is no definitive conclusion about the responsivity stability of the AES instrument. The instrument was stable over a short time using the NIST standard lamp, as shown in Fig. 5.8 (a). However, over a longer time, the responsivity either increased or decreased, depending upon the lamp used, as shown in Fig. 5.9 (a). Therefore, either the instrument or the lamps were not stable, the latter possibility being consistent with the relative difference shown in Fig. 5.7 (b) for lamp S-702. The responsivity of the EPA instrument is definitely stable over several days, as shown in Fig. 5.8 (b). Thus, the changes in responsivity using the participants’ lamps shown in Fig. 5.9 (b) are due to instabilities in these lamps.

**Fig. 5.10:**
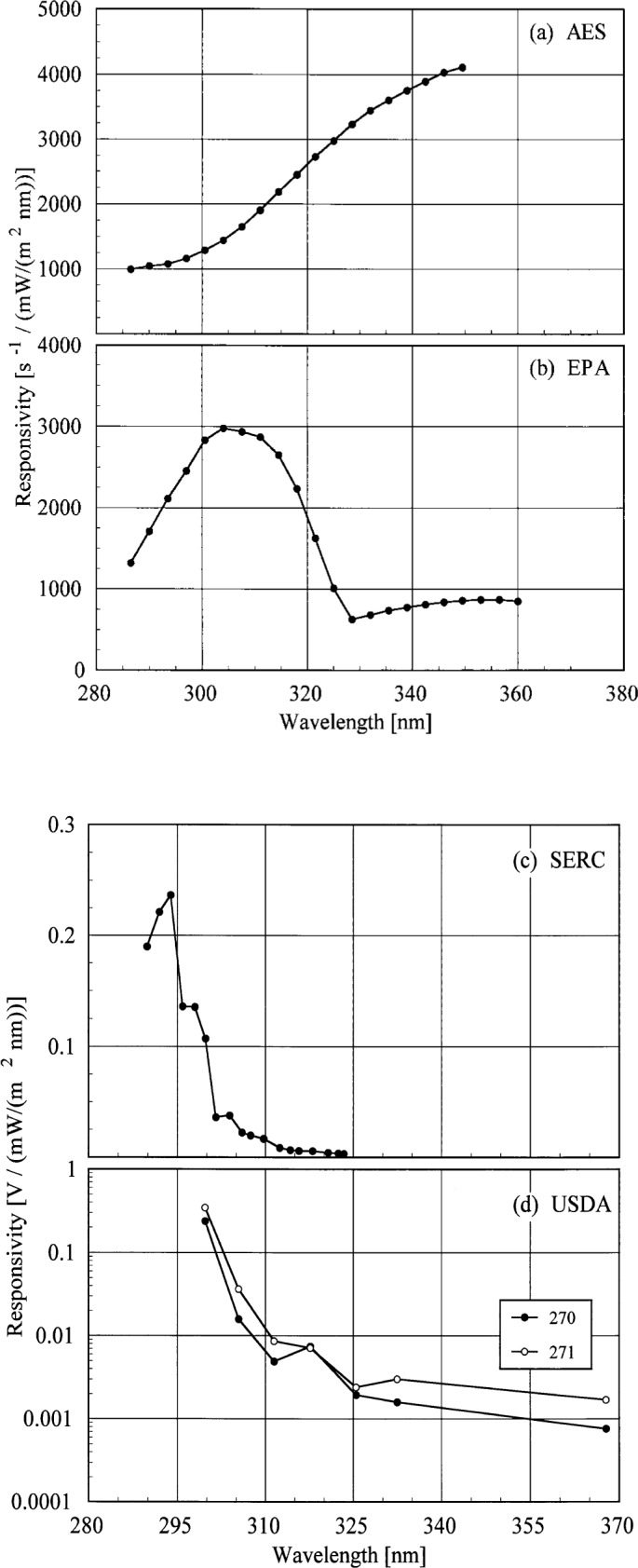
Responsivity as a function of wavelength for each instrument indicated in the panels. The USDA units are indicated in the legend in (d).

The responsivities determined using the NIST standard lamp were used to calculate the irradiances from the synchronized solar scans. Using a common standard for responsivity simplifies intercomparisons between measured irradiances because differences between spectral irradiance scales are removed from the analysis. Therefore, actual instrument performances can be evaluated more readily. The responsivities of the instruments as a function of wavelength are shown in Fig. 5.10.

## 6. Solar Irradiance

### 6.1 Introduction

The ultimate goal of the Intercomparison was to have all the instruments measure the solar ultraviolet irradiance concurrently, which was achieved over several days of the Intercomparison. The solar ultraviolet irradiance *E*(*λ*_0_) was calculated from the measured signals *S*(*λ*_0_) using the simplified measurement equation

$$E(\lambda_{0})=S(\lambda_{0})/R(\lambda_{0}),$$ (6.1)

with the responsivity *R*(*λ*_0_) for each instrument being that determined from outdoor scans of the NIST standard lamp. This was done to provide a common irradiance scale for all the instruments, thereby removing discrepancies caused by different scales and facilitating comparisons between instruments.

### 6.2 Experimental Procedure

Synchronized spectral scans of the solar ultraviolet irradiance began on the hour and half-hour from wavelengths of 290 nm to 340 nm at increments of 0.2 nm with 3 s between each wavelength. This range was common to the AES and EPA instruments. The clock for each instrument was set daily from a common clock synchronized with the satellite Global Positioning System. The synchronized scans lasted 17 min, and the maximum discrepancy in time between instruments during these scans was 2 s. Other measurements, such as wavelength calibrations and total column ozone, were performed by the AES and EPA instruments during the times between synchronized scans. The days, times, and participating instruments for the synchronized solar scans are listed in Table 6.1. The use of a neutral-density filter in the AES instrument was not resolved until 18.0 h on day 172.

### 6.3 Data Analysis

For all instruments, the measured signal was corrected before the irradiance was calculated. For the AES and EPA instruments, the signal was converted to a photon rate as detailed in [2] with dark subtraction and dead-time correction. Dark subtraction was performed for the SERC instrument.

The stray-light rejections of the instruments, shown in Fig. 5.2, can result in relatively large signals at the shortest wavelengths. To account for this, stray-light subtraction was employed for the EPA instrument. The signals at wavelengths shorter than 292 nm were averaged and subtracted from all signals from the scan. It was these signals with the stray-light subtraction that were divided by the responsivity to obtain the solar ultraviolet irradiance. The stray-light rejection of the AES instrument was sufficiently great that no correction to the signals at the shortest wavelengths was necessary.

To account for changes in responsivity over time, the responsivities of each instrument used to calculate the solar irradiance were those determined closest in time to the synchronized scans. The days and times of the responsivities used for the solar irradiances are given in Table 6.2. The responsivities of the EPA instrument were extrapolated to 325.2 nm using a third-order polynomial fit. From Eq. (6.1), the irradiance at a given wavelength is the signal at that wavelength divided by the responsivity at that same wavelength. Because the responsivities were not determined at all the wavelengths of the synchronized solar scans, the responsivities at these wavelengths were calculated from natural cubic spline interpolations.

### 6.4 Results and Discussion

**Fig. 6.1:**
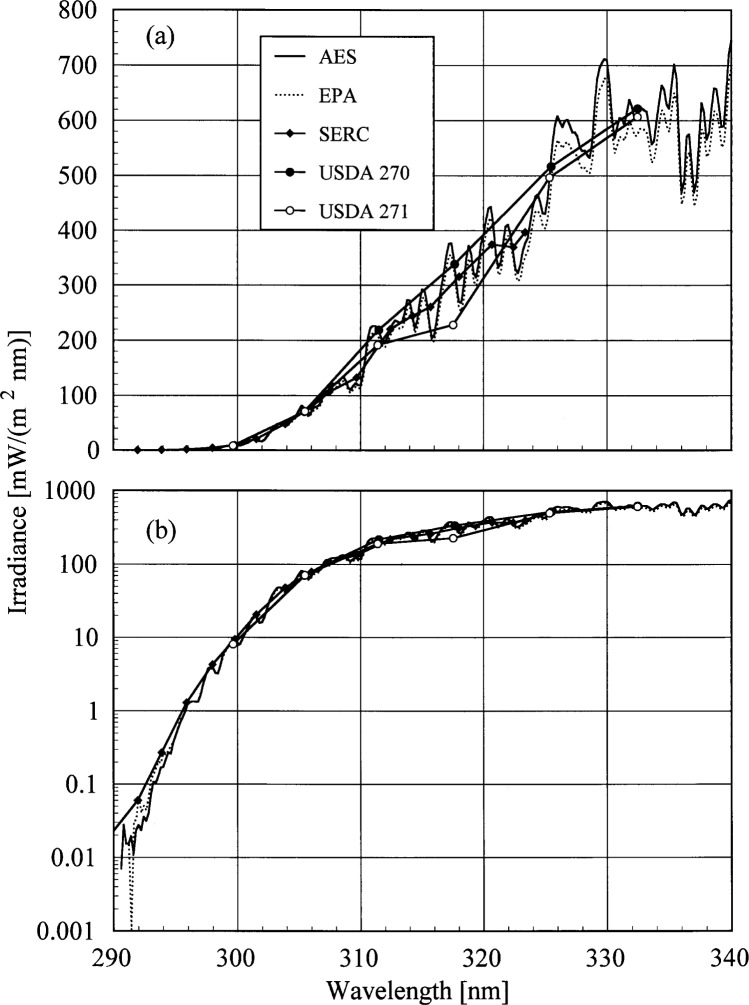
Solar irradiance on a linear scale (a) and on a logarithmic scale (b) as a function of wavelength determined by the instruments indicated in the legend on day 175 at 19.0 h

The solar irradiance measured by all instruments as a function of wavelength from a synchronized spectral scan on day 175 at 19.0 h is shown in Fig. 6.1. The irradiance is plotted on a linear scale in Fig. 6.1(a) and on a logarithmic scale in Fig. 6.1(b). The challenges encountered in accurately measuring the solar ultraviolet irradiance and of comparing the measurements between instruments, specifically wavelength accuracy, stray-light rejection, and convolution techniques, were addressed in [3].

**Fig. 6.2:**
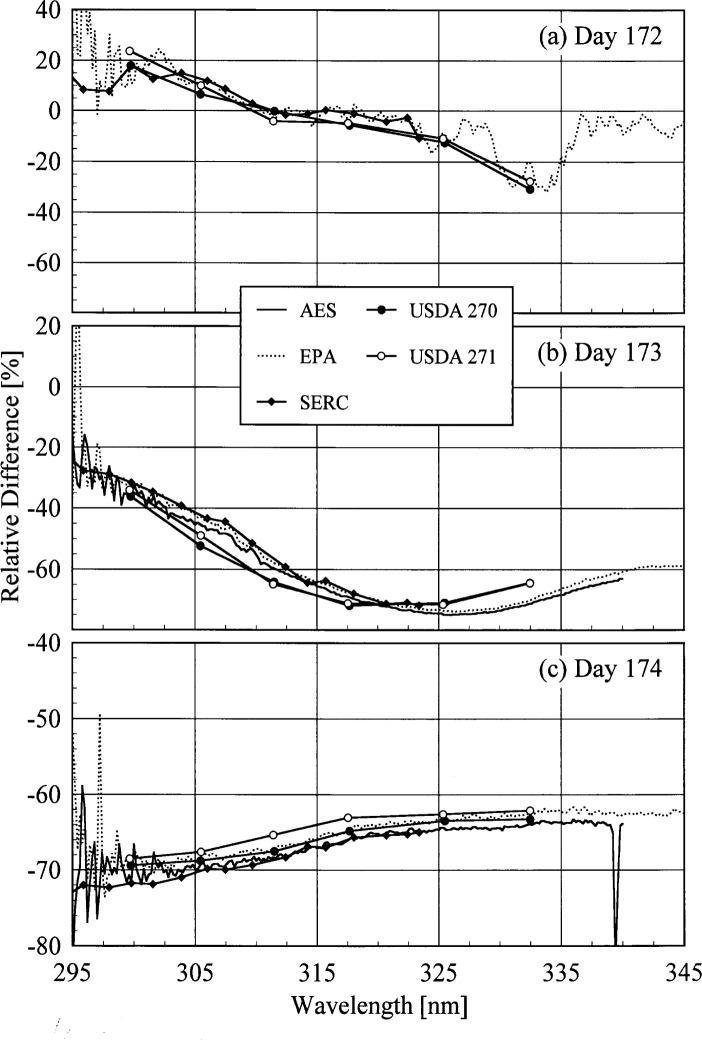
Relative difference between solar irradiances measured on the day indicated in the panel to those measured on day 175, both at 16.0 h, as a function of wavelength for the instruments indicated in the legend.

Because one goal of all the monitoring networks is to detect changes in solar ultraviolet irradiance due to ozone depletion, it is instructive to compare the irradiances measured by each instrument on different days. Because the best atmospheric conditions occurred on day 175, and all the instruments were measuring at 16.0 h on each day, the relative differences between the solar irradiances measured on other days to those measured on day 175 were calculated. The results are shown in Fig. 6.2, where the relative differences are plotted as a function of wavelength. The results for the AES instrument on day 172 are not included because it was not operating properly at 16.0 h on that day. From Fig. 6.2, the relative differences of all the instruments are consistent with each other on all the days. Also, the absence of any spectral structure in the relative differences of the AES and EPA instruments, except at the longer wavelengths on day 172 for the EPA instrument, indicate good wavelength stability for these instruments.

The results can be understood from the atmospheric conditions at the times of the measurements. The cloudy conditions on days 173 and 174, from Fig. 4.1, resulted in significantly lower irradiances of these days than on day 175, which was clear, while the irradiances on day 172 were comparable because the sky was only partly cloudy. The decrease in the relative difference as a function of wavelength on days 172 and 173 is due to the total column ozone, which from Fig. 4.2 was less on these days than on day 175. The wavelength-dependent increase in the relative difference on day 174 suggests that the total column ozone was greater on this day than on day 175.

For the previous Intercomparisons, the solar irradiances measured by the instruments were compared by convolving the irradiances with various slit-scattering functions [2, 3]. This reduced effects caused by the different bandwidths of the instruments. For comparisons between the scanning instruments, the measured irradiances were convolved with idealized rectangular, triangular, or Gaussian slit-scattering functions. To include the filter instruments in the comparisons, the irradiances measured by the scanning instruments were convolved with the filter transmittances. The value used to quantify the agreement between instruments was the standard deviation of the convolved irradiances divided by the average irradiance at each wavelength, expressed as the relative standard deviation.

Unfortunately, using the relative standard deviation to indicate the agreement between instruments is not feasible for analyzing the irradiances measured at this Intercomparison. There were only two scanning instruments operating properly and, as shown in Fig. 6.1, there is a noticeable discrepancy between the irradiances measured by these instruments. Therefore, the EPA instrument was chosen as a reference, and the irradiances measured by the other instruments were compared to those measured by this one, after the proper convolutions. For comparisons between the AES and EPA instruments, the irradiances from both instruments were convolved with a 1 nm FWHM rectangle to remove any effects from wavelength shifts. For the filter instruments, the irradiances measured by the EPA instrument were convolved with the filter transmittances of each instrument. The EPA instrument is an appropriate reference because it is a scanning instrument, similar ones were at the previous Intercomparisons, and the AES instrument was a relatively new design.

**Fig. 6.3:**
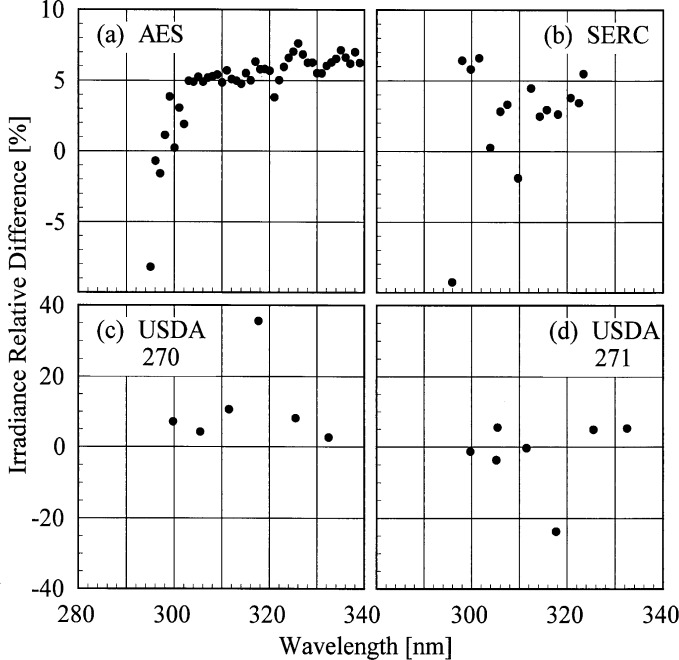
Relative difference between solar irradiances measured by the instruments indicated in the panels and the solar irradiance measured by the EPA instrument on day 175 at 19.0 h as a function of wavelength.

**Fig. 6.4:**
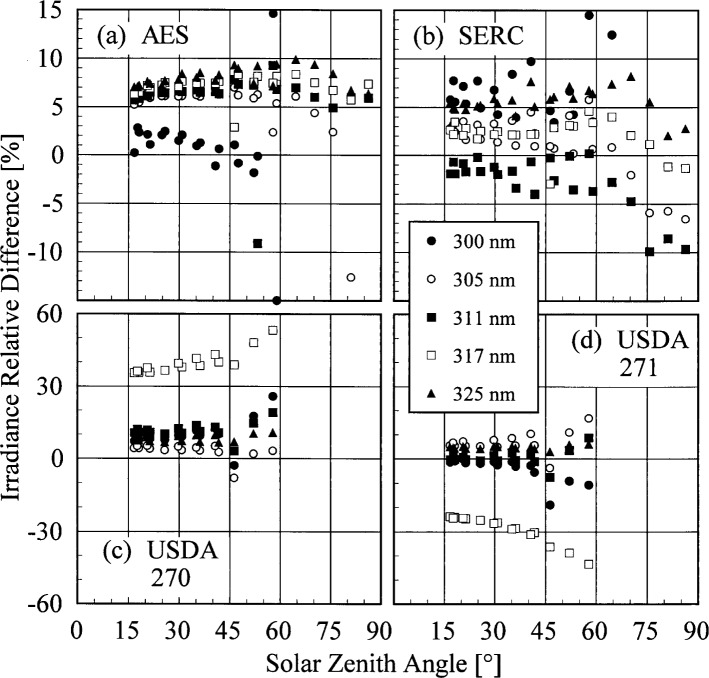
Relative difference between solar irradiances measured by the instruments indicated in the panels and the solar irradiance measured by the EPA instrument on day 175 as a function of solar zenith angle at the wavelengths indicated in the legend.

The results presented here focus on the irradiances measured on day 175 because the instruments were operating properly for most of the day and the sky was clear. The results are representative of those obtained on other days. The relative difference between the irradiances measured by each instrument to the irradiance measured by the EPA instrument as a function of wavelength at 19.0 h are shown in Fig. 6.3. To present the results for the entire day, the relative differences between the irradiances are shown as a function of solar zenith angle at selected wavelengths in Fig. 6.4.

The irradiances measured by the AES instrument were consistently greater by approximately 5 % than those measured by the EPA instrument for wavelengths longer than 300 nm. The sharp decrease in the relative difference for wavelengths shorter than 300 nm shown in Fig. 6.3 (a) is most likely due to more stray light in the EPA instrument, causing the measured irradiance to be greater. This discrepancy between the irradiances measured by the two instruments is consistent with solar zenith angle, ranging from 5 % to 10 %. This consistency implies an inherent, systematic difference between the instruments. One explanation consistent with the difference is that the greater stray light in the EPA instrument causes the responsivity to be greater, leading to a decreased measured irradiance. However, such a large discrepancy was not observed with the double monochromator instruments participating in the other Intercomparisons [2, 3]. Also, the relative differences from the other instruments are not sufficiently conclusive to determine which scanning instrument is operating incorrectly. The wavelength uncertainties of the instruments, shown in Fig. 5.4, are not sufficiently great to account for the discrepancy.

The irradiances measured by the SERC instrument were generally between −5 % and 10 % of those measured by the EPA instrument, while those measured by the USDA instruments were between 0 % and 10 % for unit 270 and – 10 % and 10 % for unit 271. The notable exceptions to this are the measured irradiances at 317 nm for the USDA instruments, which were 30 % to 60 % high for unit 270 and 25 % to 40 % low for unit 271. These differences between the irradiances measured by the USDA instruments and by the EPA instrument were consistent over the days of the Intercomparison, indicating that there was a fundamental problem with the USDA instruments at this wavelength. This is further corroborated by the relative differences shown in Fig. 6.2 for the USDA instruments, which are also consistent between days. While the relative differences remained fairly constant with solar zenith angle for wavelengths longer than 305 nm, they increased rapidly with increasing solar zenith angle at the shorter wavelengths and at 317 nm for the USDA instruments.

In summary, the measured irradiances were generally within 10 % of those measured by the EPA instrument. The 5 % to 10 % discrepancy between the irradiances measured by the AES and EPA instruments indicates an inherent problem with one of them that cannot be resolved with the results obtained at this Intercomparison. Therefore, the irradiances measured at this Intercomparison have limited usefulness for determining the best agreement that can be obtained between ultraviolet monitoring instruments. The irradiances measured by the USDA instruments were consistent with those obtained with the other instruments, except at 317 nm, where there was a definite, fundamental problem.

## 7. Conclusions

The measurements made at the 1996 Intercomparison culminated the experimental techniques developed at the previous Intercomparisons for performing all the instrument characterizations outdoors. Spectral scans of the emission lines from Cd, Hg, and Zn lamps and a HeCd laser were all performed outdoors. The stray-light rejections of the instruments were consistent with those expected for single- and double-monochromators and for interference filters. The bandwidths of the scanning instruments decreased with increasing wavelength, and their wavelength uncertainties had some variation with wavelength. All of these results were similar to those obtained at the prior Intercomparisons.

The greatest success of the Intercomparison was evaluating techniques for determining the spectral irradiance responsivity of instruments in the field. This began before the Intercomparison by measuring the spectral irradiances of participants’ lamps at NIST in both the vertical and horizontal orientations. These lamps had been calibrated prior to these measurements using the spectral irradiance scales maintained by the participants or by secondary laboratories. The results demonstrated that the participants’ scales, with the exception of the NSF, agreed with the NIST scale. Also, the spectral irradiances of the participants’ lamps in the horizontal orientation decreased relative to those when the lamps were in the vertical orientation. Therefore, while the spectral irradiance scales maintained by the participants are in agreement with the NIST scale, lamps for use in the horizontal orientation must be calibrated in this position, and additional measurements are required to remove discrepancies between the spectral irradiance scales for horizontal lamps.

The field calibration units built by NIST and NOAA were used on the first day of the Intercomparison to measure the responsivities of the EPA and SERC instruments. Because the responsivities did not depend on the unit, the units are equivalent. Similar results were obtained with the two power supply units. The spectral irradiance responsivity of each instrument was determined with a NIST standard lamp operating in a field calibration unit at least three times outdoors over the course of the Intercomparison. The responsivities of the AES and EPA instruments were also determined using the participants’ lamps. The spectral irradiance scales of the AES and EPA were within 5 % of the NIST scale. This was a greater difference than that obtained at NIST prior to the Intercomparison, indicating that these networks had difficulty either in transferring the scales to horizontal lamps used in the field or in operating the lamps. The USDA scale was markedly different from the NIST scale, most likely due to instabilities with the instruments. The responsivities of the instruments remained relatively stable for the AES and EPA instruments, while the responsivities of the SERC and USDA instruments were considerably less stable, especially for USDA unit 270.

Synchronized solar irradiance scans from 290 nm to 340 nm were performed every half-hour for four days of the Intercomparison. The results from these scans were disappointing because only two scanning instruments were operational and there was only one clear day. There was an unexplained systematic discrepancy of 5 % to 10 % between the irradiances measured by the AES and EPA instruments throughout each day, and the irradiances measured by the USDA instruments at 317 nm were consistently incorrect.

Overall, the Intercomparison was a moderate success. The instrument characterizations were correctly performed outdoors and valuable results were obtained for the participants’ spectral irradiance scales and the two different field calibration units. However, the weather was often stormy, one scanning instrument did not operate properly, and the measured solar irradiances had unexplained systematic discrepancies between instruments.

## Figures and Tables

**Table 1.1:** Instruments present during the 1996 North American Interagency Intercomparison of Ultraviolet Monitoring Spectroradiometers

Participating spectroradiometers		
Network	Instrument	Serial No.
AES	Sci-Tec Brewer MKIII	085
EPA	Sci-Tec Brewer MKIV	101
USDA	Yankee UVRSR	270 and 271
SERC	SERC SR-18	UI

Ancillary instruments	
Instrument	Serial No.
Eppley Precision Solar Pyranmometer	73-38
Eppley Precision Solar Pyranmometer	73-44
Eppley Precision Solar Pyranmometer	73–99
Eppley Precision Infrared Pyrgeometer	29143
Eppley Precision Infrared Pyrgeometer	29144
Eppley Precision Infrared Pyrgeometer	29149
Eppley Normal Incidence Pyrheliometer	16665E6
Yankee UVB-1 Radiometer	940401
Yankee UVB-1 Radiometer	940402
Yankee UVB-1 Radiometer	940404
Solar Light Biometer	1501
Solar Light Biometer	1503
Solar Light Biometer	1506
Yankee Multi-Filter Rotating Shadowband Radiometer	8709
Yankee Ultraviolet Rotating Shadowband Radiometer	10150

Meteorological instruments	
Measurement	Instrument
Temperature and relative humidity	Vaisala HMP 35C
Wind speed and direction	R. M. Young 05305
Barometric pressure	Vaisala PTB101B

**Table 3.1:** Spectroradiometer specifications

Participant	AES	EPA	USDA	SERC
Spectroradiometer				
Model	Brewer	Brewer	Yankee	SERC
	MK III	MK IV	UVRSR	SR-18
Serial No.	085	101	270, 271	UI
F-number	6	6		
Diffraction grating				
Number	2	1		
Type	plane holographic	plane holographic		
Lines per millimeter	1800	1200		
Diffraction order	second	third		
Dispersion	1 nm/mm	1 nm/mm		
Detector	9789QA	9789QA	Si	R-1657
Bandwidth (nm)	0.6	0.6	2 (nominal)	2 (nominal)
Step (nm)				
usual	0.5	0.5	2 (nominal)	2 (nominal)
finest	0.1	0.1		
Range (nm)	286 to 363	286 to 363	300 to 368	290 to 324
Diffuser material	Teflon	Teflon	Spectralon	Teflon
Weatherproof?	Yes	Yes	Yes	Yes
Automatic?	Yes	Yes	Yes	Yes
Temperature				
Stabilized optics?	No	No	No	No
Stabilized detector?	No	No	Yes	Yes
Dark current removed?	Yes	Yes	Yes	Yes
Stray light removed?	Yes	Yes	No	No
Wavelength				
registration (nm)	302.3	302.3		
Primary lamp (W)	1000	1000	1000	1000
Secondary lamp (W)	50	50		

**Table 3.2:** Channel indicator, nominal and actual center wavelength, bandwidth, and maximum transmittance for each filter of SERC instrument UI. An asterisk with the maximum transmittance indicates the channel has an additional 0.5 OD neutral-density filter

Channel	Nominal center wavelength (nm)	Actual center wavelength (nm)	Bandwidth (nm)	Maximum transmittance
A	290	289.85	2.23	0.086
B	292	291.94	2.01	0.138
C	294	293.87	2.30	0.150
D	296	295.88	2.23	0.100
E	298	297.97	2.26	0.112
F	300	299.78	2.07	0.118
G	302	301.53	2.12	0.122
H	304	303.87	2.54	0.152^*^
I	306	305.99	2.21	0.140^*^
J	Dark			
K	308	307.45	2.36	0.147^*^
L	310	309.67	2.09	0.182^*^
M	312	312.41	2.10	0.145^*^
N	314	314.22	2.28	0.131^*^
O	316	315.70	2.29	0.130^*^
P	318	318.02	2.43	0.148^*^
Q	320	320.69	2.58	0.157^*^
R	322	322.42	2.30	0.187
S	324	323.38	2.25	0.170
T	Dark			

**Table 3.3:** Channel indicator, nominal and actual center wavelength, and bandwidth for each filter of USDA instruments 270 and 271

Channel	Nominal center wavelength (nm)	Actual center wavelength (nm)	Bandwidth (nm)
Unit 270			
0	300	299.73	2.31
1	305	305.42	2.15
2	311	311.47	2.28
3	317	317.65	2.18
4	325	325.48	1.89
5	332	332.46	2.03
6	368	367.78	1.71
Unit 271			
0	300	299.64	2.28
1	305	305.47	2.15
2	311	311.39	2.34
3	317	317.54	2.11
4	325	325.35	1.95
5	332	332.46	2.11
6	368	367.62	2.28

**Table 5.1:** Dates, lamps, times, and instrument temperatures of spectral scans determining responsivity

Instrument	Day	Lamp	Time (h)	Instrument temperature (°C)
AES	171	E-004	23.5	43.2
	173	S-790	22.5	34.2
		S-790	22.7	34.2
		S-790	23.0	33.8
		S-790	23.3	33.5
	174	E-004	0.2	32.0
		S-849	17.3	25.8
		E-004	18.3	30.3
		F-370	19.6	35.7
		S-702	23.1	33.5
		S-790	23.9	33.5
	175	S-790	0.5	34.6
		S-849	1.3	33.8
EPA	171	271	22.6	39.4
		272	23.3	37.6
	172	273	0.1	36.1
		E-004	0.8	32.5
	173	E-004	22.2	29.9
		273	23.8	28.1
	174	274	0.5	26.9
		275	18.1	25.1
		271	19.1	29.6
		E-004	22.0	26.4
	176	271	18.3	30.7
SERC	171	E-004	22.3	41.8
	172	E-004	22.2	44.0
	174	E-004	17.1	29.4
		E-002	17.8	31.3
USDA 270	172	E-002	1.1	
		E-002	23.4	
	174	E-002	18.7	
USDA 271	171	E-002	23.7	
	172	E-002	22.5	
	173	E-002	23.5	
	174	E-002	18.0	

**Table 5.2:** Relative standard uncertainties from all components during responsivity measurements at selected wavelengths

Component	Wavelength (nm)	AES	Relative standard uncertainty (%)			
			EPA	SERC	USDA	USDA
Lamp						
Irradiance	290	0.92	0.92	0.92		
	320	0.90	0.90	0.90	0.90	0.90
	350	0.71	0.71		0.71	0.71
Size		0.09	0.09	0.03	0.01	0.01
Goniometry		0.46	0.46	0.27	0.11	0.11
Current (random)	290	0.06	0.06	0.06		
	320	0.05	0.05	0.05	0.05	0.05
	350	0.05	0.05		0.05	0.05
Current (systematic)	290	0.11	0.11	0.11		
	320	0.10	0.10	0.10	0.10	0.10
	350	0.09	0.09		0.09	0.09
Alignment		0.39	0.39	0.39	0.73	0.73
Instrument						
Wavelength	290	0.25	0.24	0.21		
	320	0.04	0.04	0.16	0.16	0.16
	350	0.05	0.03		0.12	0.12
Signal	290	0.94	0.08	0.23		
	320	0.35	0.53	0.46	0.96	0.60
	350	0.18	0.07		0.85	0.41
Combined						
Random	290	0.94	0.10	0.24		
	320	0.35	0.53	0.46	0.96	0.60
	350	0.19	0.09		0.85	0.41
Systematic	290	1.14	1.13	1.06		1.19
	320	1.09	1.09	1.07	1.18	1.18
	350	0.94	0.94		1.04	1.04

**Table 5.3:** Lamps, times, and temperature changes between measurements for responsivity ratios used in the figures

Numerator				Denominator			
Figure	Lamp	Day	Time (h)	Lamp	Day	Time (h)	Temperature change (°C)
5.7(a)	E-004	174	0.2	S-790	173	22.5	−2.2
5.7(a)	E-004	174	0.2	S-790	173	23.0	−1.8
5.7(a)	E-004	174	18.3	S-790	175	0.5	−4.3
5.7(b)	E-004	174	18.3	S-849	174	17.3	+ 4.5
5.7(b)	E-004	174	18.3	S-702	174	23.1	−3.2
5.7(b)	E-004	174	18.3	S-849	175	1.3	−3.5
5.7(c), (e)	E-004	172	0.8	271	171	22.6	−6.9
5.7(c), (e)	E-004	172	0.8	272	171	23.3	−5.1
5.7(c), (e)	E-004	172	0.8	273	172	0.1	−3.6
5.7(d), (f)	E-004	174	22.0	271	174	19.1	−3.2
5.7(d), (f)	E-004	173	22.2	273	173	23.8	+ 1.8
5.7(d), (f)	E-004	173	22.2	274	174	0.5	+ 3.0
5.7(d), (f)	E-004	174	22.0	275	174	18.1	+ 1.3
5.8(a)	E-004	174	18.3	E-004	174	0.2	−1.7
5.8(b)	E-004	173	22.2	E-004	172	0.8	−2.6
5.8(b)	E-004	174	22.0	E-004	173	22.2	−3.5
5.8(c)	E-004	172	22.2	E-004	171	22.3	+ 2.2
5.8(c)	E-004	174	17.1	E-004	172	22.2	−14.6
5.8(d)	E-002	172	23.4	E-002	172	1.1	
5.8(d)	E-002	174	18.7	E-002	172	23.4	
5.8(e)	E-002	172	22.5	E-002	171	23.7	
5.8(e)	E-002	173	23.5	E-002	172	22.5	
5.8(e)	E-002	174	18.0	E-002	173	23.5	
5.9(a)	S-790	173	23.0	S-790	173	22.5	−0.4
5.9(a)	S-790	174	23.9	S-790	173	23.0	−0.3
5.9(a)	S-849	175	1.3	S-849	174	17.3	+ 8.0
5.9(b)	271	174	19.1	271	171	22.6	−9.8
5.9(b)	273	173	23.8	273	172	0.1	−8.0

**Table 6.1:** Days, times, and participating instruments of synchronized spectral scans of solar ultraviolet irradiance

Participating instruments						
Day	Time (h)	AES	EPA	SERC	USDA 270	USDA 271
172	11.0			X		
	11.5			X		
	12.0			X	X	X
	12.5			X	X	X
	13.0			X	X	X
	13.5		X	X	X	X
	14.0		X	X	X	X
	14.5		X	X	X	X
	15.0		X	X	X	X
	15.5		X	X	X	X
	16.0		X	X	X	X
	16.5		X	X	X	X
	17.0		X	X	X	X
	17.5		X	X	X	X
	18.0	X	X	X	X	X
	18.5	X	X	X	X	X
	19.0	X	X	X	X	X
	19.5	X	X	X	X	X
	20.0	X	X	X	X	X
	20.5	X	X	X	X	X
	21.0	X		X	X	X
	21.5			X	X	X
	22.0			X		
	22.5			X		
	23.0			X		X
	23.5			X		X
173	13.0	X	X			
	13.5	X	X			
	14.0	X	X			X
	14.5	X	X		X	X
	15.0	X	X		X	X
	15.5	X	X	X	X	X
	16.0	X	X	X	X	X
	16.5	X	X	X	X	X
	17.0	X	X	X	X	X
	17.5	X	X	X	X	X
	18.0	X	X	X	X	X
	18.5	X	X	X	X	X
	19.0	X	X	X	X	X
	19.5	X	X	X	X	X
	20.0	X	X	X	X	X
	20.5	X	X	X	X	X
	21.0	X	X	X	X	X
	21.5			X	X	X
	22.0			X	X	X
	22.5			X	X	X
	23.0			X	X	X
	23.5			X	X	X
174	13.0	X	X	X		
	13.5	X	X	X		
	14.0	X	X	X	X	X
	14.5	X	X	X	X	X
	15.0	X	X	X	X	X
	15.5	X	X	X	X	X
	16.0	X	X	X	X	X
	16.5	X	X	X	X	X
	17.0		X		X	X
	17.5				X	X
	18.0				X	X
	18.5					
	19.0					
	19.5					
	20.0					
	20.5					
	21.0	X				
	21.5	X				
	22.0	X				
175	11.0	X	X	X		
	11.5	X	X	X		
	12.0	X	X	X		
	12.5	X	X	X		
	13.0	X	X	X		
	13.5	X	X	X		
	14.0	X	X	X		
	14.5	X	X	X		
	15.0	X	X	X		
	15.5	X	X	X		
	16.0	X	X	X	X	X
	16.5	X	X	X	X	X
	17.0	X	X	X	X	X
	17.5	X	X	X	X	X
	18.0	X	X	X	X	X
	18.5	X	X	X	X	X
	19.0	X	X	X	X	X
	19.5	X	X	X	X	X
	20.0	X	X	X	X	X
	20.5	X	X	X		
	21.0	X	X	X	X	X
	21.5	X	X	X	X	X
	22.0	X	X	X	X	X
	22.5	X	X	X	X	X
	23.0	X	X	X	X	X
	23.5	X	X	X	X	X
176	0		X	X	X	X
	0.5		X	X	X	X
	1.0		X	X	X	X
	1.5		X	X	X	X
	2.0		X	X	X	X
	2.5		X	X		
	3.0		X	X		

**Table 6.2:** Days and times of responsivity scans used to calculate solar irradiances

Day/Time of responsivity scan					
Day of solar scan	AES	EPA	SERC	USDA 270	USDA 271
172	174/0.2	172/0.8	172/22.2	172/23.4	172/22.5
173	174/0.2	173/22.2	172/22.2	172/23.4	173/23.5
174	174/18.3	174/22.0	174/17.1	174/18.7	174/18.0
175	174/18.3	174/22.0	174/17.1	174/18.7	174/18.0

## Footnotes

1 Certain commercial equipment, instruments or materials are identified in this paper to foster understanding. Such identification does not imply recommendation or endorsement by the National Institute of Standards and Technology, nor does it imply that the materials or equipment identified are necessarily the best available for the purpose.

## 8. Appendix A. Attendees

The following people attended the 1996 North American Interagency Intercomparison of Ultraviolet Monitoring Spectroradiometers, and are grouped by function and network.

**Table:**

Coordinators		
National Institute of Standards and Technology (NIST)		
Ambler Thompson	(301) 975-2333	ambler.thompson@nist.gov
Ted Early	(301) 975-2343	edward.early@nist.gov
Carol Johnson	(301) 975-2322	cjohnson@nist.gov
Fax	(301) 840-8551	
NIST		
Bldg. 220, Rm. A-320		
Gaithersburg, MD 20899		
National Oceanic and Atmospheric Administration (NOAA)		
John DeLuisi	(303) 497-6083	deluisi@srrb.noaa.gov
Patrick Disterhoft	(303) 497-6355	dister@srrb.noaa.gov
Fax	(303) 497-6546	
NOAA R/E/ARx1		
325 Broadway		
Boulder, CO 80303		
Participants		
Atmospheric Environment Service (AES)		
David Wardle	(416) 739-4632	dwardle@dow.on.doe.ca
Edmund Wu	(416) 739-4256	ewu@dow.on.doe.ca
Fax	(416) 739-4281	
Environment Canada		
4905 Dufferin Street		
Toronto, ON M3H 5T4 Canada		
Environmental Protection Agency (EPA)		
Wanfeng Mou	(706) 542-6768	wmou@hal.physast.uga.edu
Fax	(706) 542-2492	
University of Georgia		
Dept. of Physics and Astronomy		
University of Georgia		
Athens, GA 30602		
National Science Foundation (NSF)		
James Ehramjian	(619) 686-1888	jime@biospherical.com
John Tusson	(619) 686-1888	tusson@biospherical.com
Tanya Mestechkina	(619) 686-1888	tanya@biospherical.com
Fax	(619) 686-1887	
Biospherical Instruments, Inc.		
5340 Riley Street		
San Diego, CA 92110-2621		
Smithsonian Environmental Research Center (SERC)		
Douglass Hayes	(301) 261-4190 ext. 131	hayes@serc.si.edu
Fax	(301) 261-7954	
Smithsonian Environmental Research Center of the Smithsonian Institution		
P.O. Box 28		
Edgewater, MD 21037		
United States Department of Agriculture (USDA)		
Mark Beaubian	(413) 863-0200	yankee@sunlight.yesinc.com
Fax	(413) 863-0255	
Yankee Environmental Systems, Inc.		
Airport Industrial Park		
Turners Falls, MA 01376		
James Gibson	(970) 491-3611	jimg@nrel.colostate.edu
Fax	(970) 491-3601	
USDA UV-B Radiation Monitoring Program		
Natural Resource Ecology Laboratory		
Colorado State University		
Fort Collins, CO 80523		
